# Antidiarrheal Thymol Derivatives from *Ageratina glabrata.* Structure and Absolute Configuration of 10-Benzoyloxy-8,9-epoxy-6-hydroxythymol Isobutyrate

**DOI:** 10.3390/molecules21091132

**Published:** 2016-09-12

**Authors:** Celia Bustos-Brito, Valeria J. Vázquez-Heredia, Fernando Calzada, Lilian Yépez-Mulia, José S. Calderón, Simón Hernández-Ortega, Baldomero Esquivel, Normand García-Hernández, Leovigildo Quijano

**Affiliations:** 1 Instituto de Química, Universidad Nacional Autónoma de México, Circuito Exterior, Ciudad Universitaria, D.F. 04510, Mexico; la_campanella@comunidad.unam.mx (V.J.V.-H.); uscalder@unam.mx (J.S.C.); shernandezortega@gmail.com (S.H.-O.); baldo@unam.mx (B.E.); 2 UIM en Farmacología, 2o Piso CORCE, UMAE Hospital de Especialidades, Centro Médico Nacional Siglo XXI, IMSS, Av. Cuauhtémoc 330, Col. Doctores, México, D.F. 06725, Mexico; fercalber10@gmail.com; 3 UIM en Enfermedades Infecciosas y Parasitarias, UMAE Hospital de Pediatría, Centro Médico Nacional Siglo XXI, IMSS, Av. Cuauhtémoc 330, Col. Doctores, México, D.F. 06725, Mexico; lilianyepez@yahoo.com; 4 UIM en Genética Humana, UMAE Hospital de Pediatría, Centro Médico Nacional Siglo XXI, IMSS, Centro Médico Nacional Siglo XXI, IMSS, Av. Cuauhtémoc 330, Col. Doctores, México, D.F. 06725, Mexico; normandgarcia@gmail.com

**Keywords:** *Ageratina glabrata*, thymol derivatives, antiprotozoal and antidiarrheal properties

## Abstract

Chemical investigation of the leaves from *Ageratina glabrata* yielded four new thymol derivatives, namely: 10-benzoyloxy-8,9-dehydro-6-hydroxythymol isobutyrate (**4**), 10-benzoyloxy-8,9-dehydrothymol (**5**), 10-benzoyloxythymol (**6**) and 10-benzoyloxy-6,8-dihydroxy-9-isobutyryl-oxythymol (**7**). In addition, (8*S*)-10-benzoyloxy-8,9-epoxy-6-hydroxythymol isobutyrate (**1**), together with other two already known thymol derivatives identified as 10-benzoyloxy-8,9-epoxy-6-methoxythymol isobutyrate (**2**) and 10-benzoyloxy-8,9-epoxythymol isobutyrate (**3**) were also obtained. In this paper, we report the structures and complete assignments of the ^1^H and ^13^C-NMR data of compounds **1**–**7**, and the absolute configuration for compound **1**, unambiguously established by single crystal X-ray diffraction, and evaluation of the Flack parameter. The in vitro antiprotozoal assay showed that compound **1** and its derivative **1a** were the most potent antiamoebic and antigiardial compounds. Both compounds showed selectivity and good antiamoebic activity comparable to emetine and metronidazole, respectively, two antiprotozoal drugs used as positive controls. In relation to anti-propulsive effect, compound **1** and **1a** showed inhibitory activity, with activities comparable to quercetin and compound **9**, two natural antipropulsive compounds used as positive controls. These data suggest that compound **1** may play an important role in antidiarrheal properties of *Ageratina glabrata*.

## 1. Introduction

*Ageratina glabrata* (Kunth) R.M. King & H. Rob., Asteraceaea, is a shrub endemic to Mexico widely distributed throughout the country. The importance of *A. glabrata* in Mexican traditional medicine is indicated by its use for treating pain and gastrointestinal disorders associated with bacterial infections. However, chemical studies of the species are scarce. Previous phytochemical studies of *A. glabrata* show that its non-polar solvent extracts are composed mainly by thymol and eudesmane derivatives [1,2,3] while the essential oil is constituted mainly by monoterpenes [4]. On the other hand, evaluation for antibacterial activity of extracts of *A. glabrata* against antibiotic resistant pathogenic bacteria showed that the non-polar extract is the most active [5]. Preliminary studies on analgesic effect of extracts of *A. glabrata* in the hot plate test showed a moderate effect [6].

As part of our search for antiprotozoal and antidiarrheal compounds in *Ageratina* species, we have previously published our results on *A. cylindrica* [7,8]. The aim of this paper is to report on the isolation, structural characterization, and the antiprotozoal and antipropulsive evaluation of the thymol derivatives **1**–**4** and **7**. Compounds **4**–**7** are new thymol derivatives, while compounds **1**–**3**, have been previously reported from the same species, although their data were poorly described [3]. The chemical structures of the compounds isolated were established by spectroscopic methods, mainly MS and 1D, 2D NMR experiments (DEPT, COSY, NOESY, HSQC, HMBC), while the structure and absolute configuration of **1**, were confirmed by single crystal X-ray diffraction.

## 2. Results and Discussion

**Figure 1:**
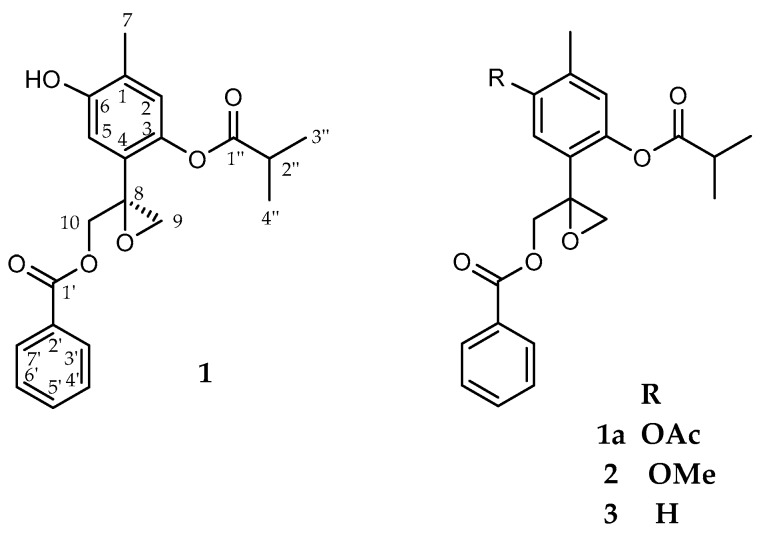
Chemical structure of **1**–**3**.

Detailed investigation of a dichloromethane extract of the leaves of *A. glabrata* resulted in the isolation of seven thymol derivatives, together with the known flavonoid pectolinaringenin [9] and benzoic acid. Compound **1** (Figure 1) was isolated as colorless crystals, whose molecular composition was determined as C_21_H_22_O_6_ on the basis of its HRDARTMS molecular ion at *m*/*z* 371.15003 [M + H]^+^ (calculated for C_21_H_23_O_6_ 371.14946), indicating nine degrees of unsaturation in the molecule. Its IR spectrum showed characteristic absorptions for hydroxyl (3599 cm^−1^) and ester groups (1757, 1723 cm^−1^).

The ^13^C-NMR spectrum of **1** showed 19 resonances representing 21 carbon atoms due to three CH_3_, two CH_2_, eight CH groups (included two symmetric ones), and eight quaternary C atoms, according to DEPT and HSQC experiments. The ^1^H-NMR spectrum exhibited in the aromatic region two one-proton signals as singlets at δ 6.79 and 6.94 assigned to H-2 and H-5, respectively, indicating a tetra-substitution of the thymol ring. Characteristic resonances in the aromatic region at δ 7.97 (2H), 7.55 (1H) and 7.40 (2H), indicated the presence of a benzoate moiety, while a heptet δ 2.83 (1H, *J* = 7.2 Hz) and two doublets at δ 1.312 and 1.307 (3H, *J* = 7.2 Hz) revealed the presence of an isobutyrate group in the molecule. Two AB spin systems with doublets at δ 3.12, 2.86 (*J* = 5.2 Hz) and 4.77, 4.48 (*J* = 12.3 Hz) due to the C-9 and C-10 methylene protons supported the presence of the oxirane and the benzoate ester functionalities, respectively.

The relative position of the ester groups was established via correlations observed in the HMBC and NOESY experiments, indicating that the benzoate was attached at C-10 and isobutyrate at C-3. Accordingly the C-10 methylene protons at δ 4.77 and 4.48 showed a ^3^*J* correlation peak with the carbonyl carbon at δ 166.3, which in turn showed correlation peaks with the aromatic protons signal at δ 7.97 (H-3’/7’) in the HMBC spectrum. On the other hand the NOESY experiment showed interactions between the isobutyrate methyl protons (H-3”, H-4”) and the methylene protons at C-9, and C-10, while the aromatic proton H-2 showed couplings with H-3”, H-4”, and the methyl protons at C-7. Therefore the isobutyrate group must be attached at C-3. Further HMBC long range couplings of the C-10 methylene protons with the quaternary carbon at δ 57.3 (C-8) and the methylene carbon at δ 51.3 (C-9), suggested the presence of the oxirane group at C-8–C-9. Acetylation of **1** with pyridine and acetic anhydride led to the acetate derivative **1a** (Figure 1). The ^1^H and ^13^C-NMR spectra (Table 1) of **1a** displayed features similar to those of **1**, except for the presence of a sharp methyl singlet at δ 2.31 in the ^1^H-NMR spectrum, and two extra carbon resonances (δ 168.9 and 20.8) in the ^13^C-NMR spectrum, associated with the presence of the acetate group. Based on all above data, the structure of compound **1**, was established as 10-benzoyloxy-8,9-epoxy-6-hydroxythymol isobutyrate.

**Figure 2:**
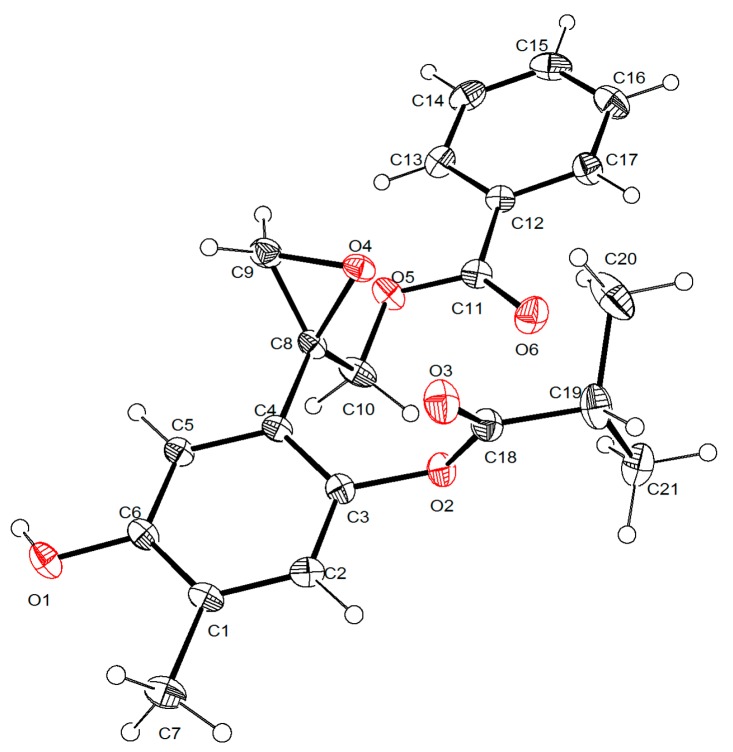
X-ray crystal structure of (+)-(8*S*)-10-benzoil-oxy-8,9-epoxy-6-hydroxy thymol isobutyrate (**1**).

Concerning the absolute configuration (AC) of chiral thymol derivatives, the only reported case is that of (8*S*)-8,9-epoxy-6-hydroxy-10-benzoyloxy-7-oxothymol isobutyrate (**9**), isolated from *A. cylindrica*. Its AC was established as 8*S* by vibrational circular dichroism spectroscopy in combination with density functional theory (DFT) calculations and evaluation of the Flack and Hooft X-ray parameters [7]. Evaluation of the Flack X-ray parameter for compound **1** defined its AC as 8*S*, consistent with that of compound **9**, with a similar structure. For this purpose a single crystal of **1** was mounted on an X-ray diffractometer equipped with CuKα monochromated radiation and collected at 150 K. Compound **1** crystallized as two crystallographically independent molecules, in the monoclinic system, space group P2(1). The structure was solved by direct methods using a full-matrix least-squares and refined to a discrepancy index of 4.23%. The absolute configuration of **1** was determined using an anomalous dispersion effects in diffraction measurements on the crystal, the Flack parameter [10], which for the enantiomer shown in Figure 2 was x = −0.03(15), thus permitting confirmation of the proposed structure, and definition of the (8*S*) configuration (Figure 2). In the structure the oxirane ring forms a dihedral angle of 56.3(2)° and 55.7(2)° with the phenol group in both structures. Crystallographic data (excluding structure factors) have been deposited with the Cambridge Crystallographic Data Centre under the reference number CCDC 1490853 contains the supplementary crystallographic data for this paper. These data can be obtained free of charge via http://www.ccdc.cam.ac.uk/conts/retrieving.html (or from the CCDC, 12 Union Road, Cambridge CB2 1EZ, UK; Fax: +44 1223 336033; E-mail: deposit@ccdc.cam.ac.uk).

It is necessary to point out that IR and ^1^H-NMR data of compound **1**, were similar to those published for eupaglabrin, a thymol derivative with an odd structure isolated from the same species [1]. Its structure was established based mainly on chemical degradation and low resolution 60 MHz ^1^H-NMR data. Years later, a compound with the structure **1** and similar spectroscopic data, was also published. Nonetheless, spectroscopic data were limited to incomplete ^1^H-NMR and some interchanged assignments [3]. In conclusion, the chemical structure and the absolute configuration of the thymol derivative previously reported from *A. glabrata* [1,3], must be represented by the stereostructure **1**.

Compound **2** (Figure 1), was isolated in minute quantities, and identified as the methyl ether derivative of **1**. Its HRDARTMS showed a pseudo-molecular ion peak at *m*/*z* 385.16429 [M + H]^+^, in agreement with a molecular formula C_22_H_24_O_6_. (calculated for C_22_H_25_O_6_, 385.16511). The NMR spectroscopic data of compound **2** (Table 2) were similar to those of **1**, except for de presence of the methoxy group with signals at δ_H_ 3.82 and δ_C_ 55.9 in its ^1^H and ^13^C-NMR spectra, respectively, in addition to the downfield shift of the C6 signal from 152.1 to 155.6 ppm, indicative of the metoxy group at that position. Thus, compound **2** was identified as the methyl ether derivative of compound **1**. Methylation of **1**, using iodomethane afforded compound **2**, spectroscopically identical with the isolated compound, allowing the complete NMR assignments.

Spectroscopic data indicated that compound **3** (Figure 1) corresponded to the 6-deoxy derivative of **1**. Its molecular composition C_21_H_22_O_5_ was deduced on the basis of its HRDARTMS pseudo-molecular ion peak at *m*/*z* 355.15516 [M + H]^+^, (calculated for C_21_H_23_O_5_, 355.15455), and ^13^C-NMR, DEPT, and HSQC experiments. The ^1^H-NMR spectrum (Table 2), showed in the aromatic region three one-proton resonances at δ 6.88 (brd, *J* = 0.7 Hz), 7.43 (d, *J* = 7.8) and 7.07 (ddd, *J* = 7.8, 1.6, 0.7 Hz) assigned to H-2, H-5 and H-6 of the thymol moiety, respectively, indicating a trisubstitution pattern of the thymol ring.

Careful analysis of the spectrum indicated long range ^1^H/^1^H couplings of H-2 and H-6 with the benzylic protons of the methyl group C-7 (^3^*J* = 1.6, ^4^*J* = 0.7 Hz). The presence of the benzoate and isobutyrate esters, were evident by the presence of characteristic benzoate signals (δ_H/C_ 7.98/129.7, 7.42/128.4 and 7.55/133.1), and the methine septet (δ_H/C_ 2.85/34.2), and methyl doublets (δ_H/C_ 1.32/18.9, 1.33/19.0) of the isobutyrate. The position of the ester moieties, was confirmed via HMBC ^2^*J* and ^3^*J* long range couplings.

**Figure 3:**
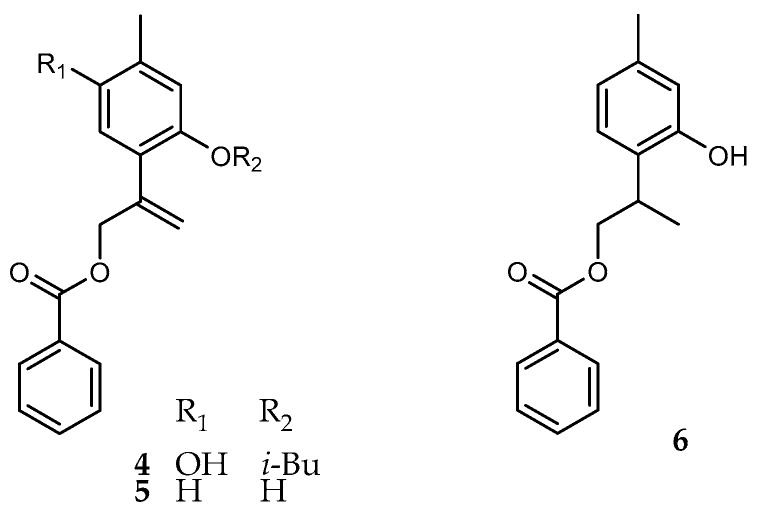
Chemical structures of **4**–**6**.

Compound **4** (Figure 3) was isolated as colorless oil, whose molecular formula C_21_H_22_O_5_ was established according with its HRDARTMS, which shows an [M + H]^+^ exact mass peak at *m/z* 355.15360 (calculated for C_21_H_23_O_5_, 355.15455). The ^1^H-NMR of compound **4** (Table 3) displayed characteristic signals for the benzoate (δ_H/C_ 8.03/130.2, 7.43/128.6 and 7.55/133.2) and the isobutyrate (δ_H/C_ 2.75/34.2, 1.27/19.1) esters. Two one-proton aromatic signals, which appeared as broadened singlets, at δ 6.81, 6.74, suggested a tetra-substituted thymol ring. The presence of two vinylic proton signals which appear as apparent quartets at δ 5.48 (*J* = 1.6 Hz, H-9a) and 5.25 (*J* = 1.2 Hz, H-9b), indicated that the common epoxy group at C-8–C-9 in compounds **1**–**3**, was replaced by an olefinic terminal methylene. The observed small *J* values, are due to alyllic couplings with the methylene protons at C-10, which appeared as a broadened doublet of doublets at δ 5.0 (*J* = 1.6, 1.2 Hz). The above data, allowed the identification of compound **4** as 10-benzoyloxy-8,9-dehydro-6-hydroxy-thymol isobutyrate (**4**), which could be considered as the biosynthetic precursor of compound **1**.

A compound with the same structure as **4** was reportedly isolated from *A. glabrata* by Bohlman et al. [3], but the limited published ^1^H-NMR data differ completely from those of compound **4**, and are in disagreement with the structure.

Compounds **1**–**3** were previously isolated from *A. glabrata* [1,3,11], but as mentioned before, only limited NMR data were available. In this paper the structure and absolute configuration of compound **1**, have been unambiguously established by NMR and single crystal X-ray diffraction, as well as, the complete assignment of the ^1^H and ^13^C-NMR data for compounds **1**–**3**.

Compounds **5** and **6** (Figure 3) are new natural thymol derivatives; they were obtained in minute amounts after repeated column chromatography and purification by thin layer chromatography. The ^1^H-NMR spectrum of **5** (Table 3) displayed similar features than those of compound **4**, except for the lack of signals ascribed to the isobutyrate moiety and the -OH group at C-6. The presence of an olefinic terminal methylene was evident from the two one-proton signals at δ 5.56 (q, *J* = 1.6, H-9a) and 5.31 (q, *J* = 1.2, H-9b), both coupled with the allylic methylene protons at C-10. Thus, the structure of compound **5** was established as 10-benzoyloxy-8,9-dehydrothymol (**5**).

Compound **6**, was identified as the dihydroderivative of **5**. Therefore, the ^1^H-NMR spectrum (Table 3), did not show the olefinic methylene signals, instead of that, the spectrum displayed a methyl doublet signal at δ 1.42 (d, *J* = 6.8 Hz, 3H) as the part X_3_, of the spin system ABMX_3_, due to H_2_-10, H-8 and H_3_-9 protons. One methine proton signal at δ 3.50 appeared as a doublet of quartets of doublets (*J* = 8.0, 6.8, 4.8, Hz, 1H), coupled with the methyl doublet and the methylene protons H_2_-10, according to the HSQC and HMBC experiments. The protons of the C-10 methylene bearing the benzoyloxy group appeared as the AB part of the spin system with signals at δ 4.19 (dd, *J* = 10.8, 8.0 Hz) and 4.53 (dd, *J* = 10.8, 4.8 Hz). Consequently, the structure of compound **6**, was established as 10-benzoyloxy thymol (**6**), and represents a new natural thymol derivative.

**Figure 4:**
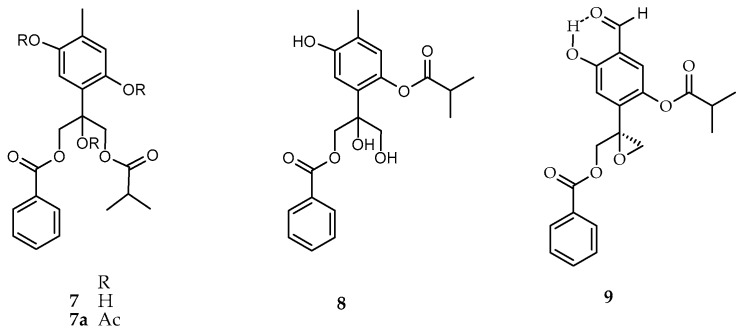
Chemical structures of **7**–**9**.

Compound **7** (Figure 4) was obtained as colorless oil, whose ^1^H and ^13^C-NMR spectra (Table 4) displayed similar spectral features as compound **1**. Two one-proton aromatic singlets at δ 6.66 and 6.60 indicating the tetrasubstitution of the thymol ring. The presence of a benzoate and an isobutyrate was evident from their characteristic signals (δ_H/C_ 7.98/129.9, 7.41/128.7, 7.55/133.6), for the benzoate and (δ_H/C_ 2.53/34.1, 1.05, 1.08/18.9, 19.0) due to the isobutyrate. The main differences between **1** and **7** were the multiplicity and chemical shifts of signals due to the methylene groups bearing the oxygen functionalities. The ^1^H-NMR spectrum displayed two AB spin systems with signals centered at δ_H_ 4.51, 4.58 (d, *J* = 11.6, C-9), and 4.64, 4.68 (d, *J* = 12.0, C-10), coupled with the methylene carbon signals at δ_C_ 67.5 and 68.1, according to the HSQC spectrum, respectively. In the HMBC experiment, the proton signals of both AB methylene groups, showed ^2^*J* and ^3^*J* long range couplings with the tertiary carbon signals at δ_C_ 78.3 (C-8) and 120.3 (C-4). On the other hand the AB system centered at δ 4.55 was coupled to the carbonyl at δ 177.9, which in turn, was coupled with the methine heptet and methyl doublets of the isobutyrate (δ_H/C_ 2.53/34.1, 1.05, 1.08/19.0), while the one, centered at δ 4.66 showed couplings with the carbonyl at δ 167.1, that in turn was, coupled with the benzoate protons H-3’/7’ (δ_H/C_ 7.98/129.9). Thus, the isobutyrate is attached to C-9, and the benzoate at C-10. According with the above data, the structure of compound **7** was established as 10-benzoyloxy-6,8-dihydroxy-9-isobutyryloxythymol (**7**).

Regarding the AC of compounds **6** and **7**, the optical rotation and ECD values of compound **6** and **7** were similar to those of compounds **1** and **9** with established 8*S* configuration, suggesting the same configuration for compounds **6** and **7**. Further investigation is nevertheless desirable in order to confirm the above assumption.

The HRDARTMS of **7**, did not show the C_21_H_25_O_7_ [M + H]^+^ ion at *m*/*z* 389, instead of that, it displayed the [M − H_2_O + H]^+^, ion peak at *m*/*z* 371.14833 in agreement with a molecular formula C_21_H_23_O_6_ (calculated 371.14946), due to the loss of water from the molecular ion. It is documented that some of these compounds are artifacts originated by the hydrolytic opening of the oxirane-ring followed by transesterification from O-3 to O-9 during the isolation process, or after purification [12,13]. In case of compound **7**, it is indeed an artifact, since it was obtained from compound **1,** after storing at room temperature for some time.

Recently a thymol derivative isolated from *A. glabrata* has been described [14]. Its proposed structure **8**, was established on the basis of MS, ^1^H and ^13^C-NMR data. Comparison of the published data with those of compound **7**, showed to be identical. Therefore, the structure **8** must be revised, and changed to the structure **7** (Figure 4).

Acetylation of **7** with pyridine and acetic anhydride yielded compound **7a** (Figure 4). The ^1^H and ^13^C-NMR spectra (Table 4) of **7a** displayed features similar to those of **7**, except for the presence of three sharp methyl singlet at δ 2.00, 2.31, 2.38 in the ^1^H-NMR spectrum, which showed couplings with the methyl carbon signals at δ 21.4, 20.9 and 21.3 in the HSQC experiment, and with the carbonyl carbons at 168.8, 168.8 and 169.4, in the HMBC experiment, respectively. In addition, downfield shifts of the aromatic protons H-2, H-5 and the methylene protons of CH_2_-9 and CH_2_-10, inherent to the presence of the acetyl groups, were observed.

Compounds **1**–**4**, **7** and **1a** were investigated for antiprotozoal activity (Table 5) against *Entamoeba histolytica* and *Giardia lamblia*. Compound **1** and its derivative **1a** showed selectivity and good antiprotozoal activity on *Entamoeba histolytica* trophozoites, being their effects similar to emetine and metronidazole, two antiprotozoal drugs used as controls. In contrast, in the case of *Giardia lamblia* both compounds showed moderate antigiardial activity. The remaining compounds **2**–**4**, **7**, and pectolinaringenin showed moderate antiprotozoal activity on both protozoa.

Compounds **1**–**4**, **7** and **1a** were also tested on the charcoal-gum acacia-induced hyperperistalsis model in rats. Compounds **1** and **4** showed moderate inhibitory activity on hyperpropulsive movement of the small intestine in rats with activities comparable to quercetin (Table 5). In addition, the remaining compounds **1a**–**3**, **7**, and pectolinaringenin showed high inhibitory activity but lower that of loperamide hydrochloride, antidiarrheal drug used as positive control. It is important to point out that the presence of an acetate group at the C-6 position in thymol derivative **1a** seems to be important for the inhibition of hyperperistalsis and antiprotozoal activity. Finally, the antidiarrheic properties reputed for *Ageratina glabrata* in Mexican traditional medicine may be due to the presence of thymol derivatives **1**–**4**, **7** and the flavonoid, pectolinaringenin.

## 3. Materials and Methods

### 3.1. General Procedures

Melting points were measured on a Fisher-Johns apparatus (Fisher Scientific Company, Pittsburgh, PA, USA) and are uncorrected. Optical rotations were measured on a 323 polarimeter (Perkin Elmer Inc., London, UK). Ultraviolet absorptions were recorded on a UV 160U spectrophotometer (Shimadzu, Kyoto, Japan). IR spectra were obtained on a Tensor 27 spectrometer (Bruker, Ettlingen, Germany). The 1D and 2D NMR experiments were performed on a Bruker Advance III spectrometer (Bruker) at 400 MHz for ^1^H and 100 MHz for ^13^C. Chemical shifts were referenced to TMS and *J* values are given in Hz. The HRDARTMS data were recorded on an AccuTOF JMS-T100LC mass spectrometer (Jeol Ltd., Tokyo, Japan). Prep TLC was carried out on precoated Sil G/UV254 plates (Macherey Nagel, Düren, Germany) of 1.0 mm thickness. Silica gel 230–400 mesh (Macherey-Nagel), Sephadex LH-20 (Pharmacia Biotech AB, Uppsala, Sweden) and octadecyl functionalized silica gel (Sigma Aldrich, St. Louis, MO, USA) were used for column chromatography. The X-ray data were collected on a D8 Venture κ-geometry diffractometer (Bruker).

### 3.2. Plant Material

*Ageratina glabrata* was collected at Cuernavaca, Morelos, Mexico, in February 2015. Plant material was identified by Oscar Hinojosa Espinoza, and a voucher specimen (MEXU-1 437 258) was deposited at the National Herbarium (MEXU) of the Instituto de Biologia, UNAM.

### 3.3. Extraction, Isolation and Characterization

The air-dried and powdered leaves of *A. glabrata* (690 g) were extracted CH_2_Cl_2_ (2 L × 3 times) at room temperature for 48 h. The extract was concentrated at reduced pressure to yield 18 g of residue. The crude extract was subjected to column chromatography (CC) on silica gel using gradient elution with EtOAc–hexanes (80:20) to obtain 29 eluates, 150 mL each, which were combined into 17 major fractions (A–Q) by TLC evaluation. Pectolinarigenin (50.8 mg) crystalized from fraction N. Fraction B (2.78 g) was subjected to CC on silica gel using gradient elution with EtOAc–hexanes (90:10) to obtain 55 eluates, 150 mL each, which were combined in 25 major fractions (BA–BX). Compound **1** (450 mg) crystalized from fractions BG to BJ. Fraction BD (725 mg) was further fractionated over silica gel using dichloromethane as eluent to obtain six major fractions (BDA–BDF), fraction BDD (64.5 mg) was subjected to silica gel TLC eluting with hexanes–EtOAc (70:30) to give compound **2** (17.9 mg). Fraction BG (261.6 mg) was subjected to silica gel CC eluting with dichloromethane:acetone (95:5) to give six major fractions (BGA–BGF). Fraction BGB was subjected to silica gel TLC eluting with dichloromethane–acetone (99:1) to give compound **3** (4.8 mg). Fraction C (120 mg) was separated by CC on silica gel eluted with dichloromethane–acetone (0:100–100:0) to obtain seven fractions (CA–CG). Compound **5** (5.0 mg)was identified in fraction CA. Fraction B was subjected to silica gel TLC eluting with dichloromethane to obtain compound **6** (9.8 mg). Pure **4** (13.3 mg) was obtained from fraction CE. Fraction G (889 mg) was subfractionated over a Sephadex LH-20 column, using MeOH as eluent to give five fractions (GA–GE). Benzoic acid (50.6 mg) crystalized from fraction GA. Ilicic acid was obtain as pure compound from fraction GB. Fraction GC was subjected to silica gel CC using CH_2_Cl_2_ as eluent to obtain seven fractions (GCA–GCG). Pure compound **7** (24.3 mg) was obtained from fraction GCD.

*(8S)-10-Benzoyloxy-8,9-epoxy-6-hydroxythymol isobutyrate* (**1**). Colorless crystals (CH_2_Cl_2_-hexane); m.p. 110–112 °C; [α]_589_ +14.5 (c 0.001, MeOH); UV (MeOH) λ_max_ (log ε) 204 (4.71), 225 (4.52), 280 (3.30) nm; ECD (MeOH) λ_max_ (Δε): 204 (−3.2), 209 (0.2), 226 (−1.4), 253 (0.3); IR (CHCl_3_) ν_max_ 3599, 2980, 1752, 1722, 1273 cm^−1^; ^1^H and ^13^C-NMR (CDCl_3_) see Table 1; HRDARTMS *m*/*z* 371.15003 (calculated for C_21_H_22_O_6_ + H, 471.14946).

*10-Benzoyloxy-8,9-epoxy-6-acetyloxythymol isobutyrate* (**1a**). Colorless oil; [α]_589_ +14.7 (c 0.002, MeOH); UV (MeOH) λ_max_ (log ε) 203 (4.47), 252 (4.52), 273 (4.51) nm; ECD (MeOH) λ_max_ (Δε): 202 (−10.5), 224 (−2.9), 244 (0.4); IR (CHCl_3_) ν_max_ 2979, 2937, 1757, 1721, 1270 cm^−1^; ^1^H and ^13^C-NMR (CDCl_3_) see Table 1; HRDARTMS *m*/*z* 413.16040 (calculated for C_23_H_24_O_7_ + H, 413.16003).

*10-Benzoyloxy-8,9-epoxy-6-methoxythymol isobutyrate* (**2**). Colorless oil; [α]_589_ +14.6 (c 0.0015, CHCl_3_); UV (MeOH) λ_max_ (log ε) 204 (4.54), 225 (4.34), 276 ( 3.70 ) nm; ECD (MeOH) λ_max_ (Δε): 204 (−6.3), 230 (−1.1), 250 (0.2); IR (CHCl_3_) ν_max_ 2979, 2938, 1751, 1722, 1272 cm^−1^; ^1^H and ^13^C-NMR (CDCl_3_) see Table 2; HRDARTMS *m*/*z* 385.16429 (calculated for C_22_H_24_O_6_ + H, 385.16511).

*10-Benzoyloxy-8,9-epoxythymol isobutyrate* (**3**). Colorless oil; [α]_589_ −3.9 (c 0.002, CHCl_3_); UV (MeOH) λ_max_ (log ε) 203 (4.27), 221 (4.16), nm; ECD (MeOH) λ_max_ (Δε): 205 (1.1), 215 (−0.5); IR (CHCl_3_) ν_max_ 2976, 2938, 1757, 1723, 1271 cm^−1^; ^1^H and ^13^C-NMR (CDCl_3_) see Table 2; HRDARTMS *m*/*z* 355.15516 (calculated for C_21_H_22_O_5_ + H, 355.15455).

*10-Benzoyloxy-8,9-dehydro-6-hydroxythymol isobutyrate* (**4**). Colorless oil; UV (MeOH) λ_max_ (log ε) 204 (4.31), 225 (4.23), 276 (3.58) nm; IR (CHCl_3_) ν_max_ 3522, 2953, 2927, 1757, 1713, 1291 cm^−1^; ^1^H and ^13^C-NMR (CDCl_3_) see Table 3; HRDARTMS *m*/*z* 355.15360 (calculated for C_21_H_22_O_5_ + H, 355.15455).

*10-Benzoyloxy-8,9-dehydrothymol isobutyrate* (**5**). Colorless oil; ^1^H and ^13^C-NMR (CDCl_3_) see Table 3.

*10-Benzoyloxythymol isobutyrate* (**6**). white powder; m.p. 94–97 °C [α]_589_ +12.27 (c 0.002, CHCl_3_); UV (MeOH) λ_max_ (log ε) 205 (3.87), 224 (3.80),275 (3.21) nm; ECD (MeOH) λ_max_ (Δε): 203 (−4.3), 217 (0.5), 238 (−0.7), 277 (1.6); IR (CHCl_3_) ν_max_ 3599, 3400, 2970, 2928, 1752, 1714, 1279 cm^−1^; ^1^H and ^13^C-NMR (CDCl_3_) see Table 3.

*10-Benzoyloxy-6,8-dihydroxy-9-isobutyryloxythymol* (**7**). Colorless oil; [α]_589_ 0.00 (c 0.001, CHCl_3_); UV (MeOH) λ_max_ (log ε) 205 (4.96), 228 (4.89), 293 (4.40) nm; ECD (MeOH) λ_max_ (Δε): 202 (−9.3), 208 (2.0), 213 (1.2), 221 (0.5); IR (CHCl_3_) ν_max_ 3603, 3398, 2979, 2936, 1724, 1291 cm^−1^; ^1^H and ^13^C-NMR (CDCl_3_) see Table 4; HRDARTMS *m*/*z* 371.14933 [M − H_2_O]^+^ (calculated for C_21_H_23_O_6_, 371.149646). ESIMS *m*/*z* 411.

*10-Benzoyloxy-6,8-diacetyloxy-9-isobutyroyloxythymol acetate* (**7a**). Colorless oil; [α]_589_ +3.60 (c 0.001, CHCl_3_); UV (MeOH) λ_max_ (log ε) 203 (6.32), 223 (6.16), 274 (5.27) nm; ECD (MeOH) λ_max_ (Δε): 202 (−6.9), 208 (0.4), 216 (−0.6), 226 (−0.5), 241 (0.2); IR (CHCl_3_) ν_max_ 2978, 2936, 1759, 1370, 1271 cm^−1^; ^1^H and ^13^C-NMR (CDCl_3_) see Table 4; DARTMS *m*/*z* 546 [M − C_4_H_8_O_2_]^+^.

### 3.4. X-ray Crystallography of Compound ***1***

Colorless crystals of 0.376 × 0.188 × 0.098 mm^3^, with empirical formula C_21_H_22_O_6_, and Mr = 370.39, crystallized in a Monoclinic crystal system, P2**_1_**, with cell parameters a = 10.0148(8) Å, b = 7.6733(6) Å, and c = 24.814(2) Å. V = 1872.2(3) Å^3^, Z = 4, D_calcd_ = 1.314 Mg/m^3^, mμ 0.796 mm^−1^, F(000) = 784.0. Compound **1** was irradiated with CuKα radiation (λ = 1.54178 Å) on the Bruker D8 Venture κ-geometry diffractometer with microfocus X-ray source and Helios multilayer mirror as monochromator, using an APEX 3 program [15] at 150(2) K. Data reduction was achieved using the SAINT program [15] Totals of 23784 reflections were collected, from which 6748 (R_int_ = 0.0767) reflections were independent. Structure was solved using direct methods and then refined with the SHELXS and SHELXL programs [16] with full-matrix least-squares on F2, respectively. ORTEP-3 software [17] was used for the figures.

The final values S = 1.064, R_1_ = 0.0479, and wR_2_ = 0.1092 were based on 6748 reflections observed, 499 parameters. The largest different peak and hole for **1** was 0.196 and −0.161 e·Å^−3^.

### 3.5. Antiprotozoal Assays

*Entamoeba histolytica* strain HM1-IMSS used in all experiments was grown axenically at 37 °C in TYI-S-33 medium supplemented with 10% heat inactivated bovine serum. In the case of *Giardia lamblia*, strain IMSS: 8909:1 was grown in TYI-S-33 modified medium supplemented with 10% calf serum and bovine bile. The trophozoites were axenically maintained and for assays were employed in the log phase of growth. In vitro susceptibility tests were performed using a subculture method previously described [18]. Briefly, *E. histolytica* (6 × 10^3^) or *G. lamblia* (5 × 10^4^) trophozoites were incubated for 48 h at 37 °C in the presence of different concentrations (2.5–200 μg/mL) of the crude extract or pure compounds in dimethyl sulfoxide (DMSO). Each test included metronidazole (Sigma) as standard amoebicidal and giardicidal drugs, a control (culture medium plus trophozoites and DMSO), and a blank (culture medium). After incubation, the trophozoites were detached by chilling and 50 μL samples of each tube were subcultured in fresh medium for another 48 h, without antiprotozoal samples. The final number of parasites was determined with a haemocytometer and the percentages of trophozoites growth inhibition were calculated by comparison with the control culture. The results were confirmed by a colorimetric method: the trophozoites, were washed and incubated for 45 min at 37 °C in phosphate buffer saline with MTT (3-(4,5-dimethylhiazol-2-il)-2,5-diphenyl tetrazolium bromide) and phenazine methosulfate. The dye produced (formazan) was extracted and the absorbance was determined at 570 nm. The experiments were performed in duplicate for each protozoan and repeated at least three times. The in vitro results were classified as follows: if the samples displayed an IC_50_ less than 20 μM, the antiprotozoal activity was considered good, from 21 to 160 μM the antiprotozoal activity was considered moderate, from 161 to 200 μM the antiprotozoal activity was considered weak and over 200 μM/mL the samples were considered inactive. Data were analyzed using probit analysis. The percentage of trophozoites surviving was calculated by comparison with the growth in the control group. The plot of probit against log concentration was made; the best straight line was determined by regression analysis and the 50% inhibitory concentration (IC_50_) values were calculated. The regression coefficient, its level of significance (*p* < 0.05 indicates significant difference between group) and correlation coefficient were calculated and 95% CI values determined.

### 3.6. Animals

Male Sprague-Dawley rats (200–250 g) were obtained from the animal house of the IMSS. These studies were conducted with the approval of the Specialty Hospital Bio-Ethical Committee of the National Medical Center “Siglo XXI” from IMSS (Approval No.: R-2012-3601-182). Investigation using experimental animals was conducted in accordance with the official Mexican norm NOM 0062-ZOO-1999 entitled Technical specifications for the production, care and use of laboratory animals [19]. They were fasted overnight but tap water was available ad libitum until the start of the experiments.

### 3.7. Effect on Charcoal-Gum Acacia-Induced Hyperperistalsis

The method, described by Williamson et al. [20] was adopted to study the effect of the compounds on hyperperistalsis in rats. The test animals were divided into control group and test groups containing six rats in each group. Rats were treated orally with each compound (0.01, 0.1, 1.0, 10, 20, 40 mg/kg in 1 mL of a 2% dimethyl sulfoxide (DMSO) solution in water), or vehicle (1 mL of a 2% DMSO solution in water) or loperamide hydrochloride (Sigma) (0.1, 1.0, 10, 20, 40 mg/kg in 1 mL of a 2% DMSO solution in water). After 20 min, each of these animals was given 1 mL of charcoal meal (10% charcoal suspension in 5% aqueous arabic gum) by oral route. All animals were sacrificed after 30 min, the stomach and small intestine were removed and extended on a clean glass surface. The distance moved by the charcoal meal from the pylorus was measured and then expressed as a percentage of the distance from the pylorus to the caecum. After, the plot of percentage of inhibition against concentration was made; the best straight line was determined by regression analysis and the 50% inhibitory concentration (IC_50_) values were calculated. The regression coefficient, its level of significance (*p*) and correlation coefficient were calculated. The experiments were performed six times for each concentration. IC_50_ values are mean ± S.E.M. *p* < 0.05 (1–way ANOVA followed by Dunnett’s post hoc test), GraphPad Prism Version 5.03 (GraphPad Software Inc., La Jolla, CA, USA) was used.

## Figures and Tables

**Table 1:** NMR spectroscopic data (^1^H 400 MHz, ^13^C 100 MHz, CDCl_3_) of **1** and **1a**.

Position	1			1a	
	δ_C_, Type	δ_H_ (*J* in Hz)	HMBC	δ_C_, Type	δ_H_ (*J* in Hz)
1	126.2, C			132.2, C	
2	124.8, CH	6.79, d (0.8)	1, 3, 4, 6, 7	125.0, CH	6.96, brs
3	141.7, C			146.2, C	
4	127.2, C			127.9, C	
5	114.8, CH	6.94, s	1, 3, 6, 8	122.7, CH	7.23. s
6	152.1, C			147.0, C	
7	15.8, CH_3_	2.18, s	1, 2, 6	16.3, CH_3_	2.17, s
8	57.3, C			56.9, C	
9a	51.3, CH_2_	3.12, d (5.2)	4, 8, 10	51.2, CH_2_	3.11, d (5.2)
9b		2.86, d (5.2)	4, 8, 10		2.87, d (5.2)
10a	65.6, CH_2_	4.77, d (12.3)	1′, 4, 8, 9	65.9, CH_2_	4.76, d (12.2)
10b		4.48, d (12.3)	1′, 4, 8, 9		4.47, d (12.2)
1′	166.3, C			166.1, C	
2′	129.7, C			129.0, C	
3′	129.8, CH	7.97, m	1′, 5′	129.9, CH	7.97, m
4′	128.5, CH	7.40, m	3′, 5′, 7′	128.5, CH	7.42, m
5′	133.4, CH	7.55, tt (7.5, 1.3)	3′, 7′	133.3, CH	7.55, tt (7.4, 1.2)
6′	128.5, CH	7.40, m	3′, 5′, 7′	128.5, CH	7.42, m
7′	129.8, CH	7.97, m	1′, 5′	129.9, CH	7.97, m
1″	176.2, C			175.3, C	
2″	34.3, CH	2.83, hep (7.2)	1″, 3″, 4″	34.3, CH	2.84, hep (7.2)
3″, 4″	19.1, 19.2, CH_3_	1.312, 1.307 d (7.2)	1″, 2″	19.0, 19.1, CH_3_	1.31, 1.32, d(7.2)
6-OH		5.78, brs			
COCH_3_				20.8, CH_3_	2.31
COCH_3_				168.9, C	

**Table 2:** NMR spectroscopic data (^1^H 400 MHz, ^13^C 100 MHz, CDCl_3_) of **2** and **3**.

Position	2		3	
	δ_C_, Type	δ_H_ (*J* in Hz)	δ_C_, Type	δ_H_ (*J* in Hz)
1	128.6, C		140.0, C	
2	124.6, CH	6.83, brs	123.1, CH	6.88, brd (0.7)
3	141.6, C		148.7, C	
4	126.9, C		126.1, C	
5	109.9, CH	6.97, s	128.9, CH	7.43, d (7.8)
6	155.6, C		126.8, CH	7.07, ddd (7.8, 1.6, 0.7)
7	16.2, CH_3_	2.19, brd (0.4)	21.1, CH_3_	2.35, s
8	57.3, C		57.0, C	
9a	51.3, CH_2_	3.13, d (5.3)	50.9, CH_2_	3.12, d (5.3)
9b		2.87, d (5.3)		2.85, d (5.3)
10a	65.8, CH_2_	4.79, d (12.2)	65.9, CH_2_	4.76, d (12.2.)
10b		4.48, d (12.2)		4.47, d (12.2)
1′	166.1, C		165.9, C	
2′	129.9, C		129.72, C	
3′	129.8, CH	7.99, m	129.67, CH	7.98, m
4′	128.5, CH	7.43, m	128.4, CH	7.42, m
5′	133.3, CH	7.55, m	133.1, CH	7.55, tt (7.6, 1.3)
6′	128.5, CH	7.42, m	128.4, CH	7.42, m
7′	129.8, CH	7.99, m	129.67, CH	7.98, m
1″	175.9, C		175.3, C	
2″	34.3, CH	2.83, hep (7.2)	34.2, CH	2.85, hep (7.0)
3″, 4″	19.1, 19.2, CH_3_	1.31, 1.32 d(7.2)	18.9, 19.0, CH_3_	1.32, 1.33, d(7.0)
OCH_3_	55.9, CH_3_	3.82, s		

**Table 3:** NMR spectroscopic data (^1^H 400 MHz, ^13^C 100 MHz, CDCl_3_) of **4**–**6**.

Position	4		5		6	
	δ_C_, Type	δ_H_ (*J* in Hz)	δ_C_, Type	δ_H_ (*J* in Hz)	δ_C_, Type	δ_H_ (*J* in Hz)
1	125.0, C		140.2, C		138.0, C	
2	124.9, CH	6.80, brs	116.9, CH	6.78, d (0.7)	116.8, CH	6.68, d (0.8)
3	141.4, C		153.5, C		154.0, C	
4	130.9, C		122.6, C		125.3, C	
5	115.9, CH	6.74, s	129.3, CH	7.03, d (7.7)	127.2, CH	7.10, d (7.8)
6	151.6, C		121.2, CH	6.71, ddd (7.7, 1.7, 0.7)	121.6, C	6.74, ddd (7.6, 0.8, 0.4)
7	15.8, CH_3_	2.22, brs	21.4, CH_3_	2.32, s	21.1, CH_3_	2.28, s
8	140.2, C	-	141.9, C		32.4, C	3.50, dqd (8.0, 6.8, 4.8)
9a	117.3, CH_2_	5.48, q (1.6)	116.7, CH_2_	5.56, q (1.6)	16.7, CH_3_	1.42, d (6.8)
9b		5.25, q (1.2)		5.31, q ( 1.2)		
10a	66.7, CH_2_	5.0, dd (1.6, 1.2)	66.6, CH_2_	4.99, dd (1.6, 1.2)	70.2, CH_2_	4.53, d (10.9, 4.8)
10b						4.19 dd (10.9, 8.0)
1′	166.3, C		167.2, C		167.5, C	
2’	130.2, C		129.8, C		130.3, C	
3′	129.8, CH	8.03, m	129.9, CH	8.09, m	129.8, CH	8.04, m
4′	128.6, CH	7.43, m	128.7, CH	7.47, m	128.5, CH	7.56, tt (7.2, 1.6)
5′	133.2, CH	7.55, tt (7.4, 1.4)	133.6, CH	7.60, tt (7.2, 1.3)	133.2, CH	7.44, m
6′	128.6, CH	7.43, m	128.7, CH	7.47, m	128.5, CH	7.56, tt (7.2, 1.6)
7′	129.8, CH	8.03, m	129.9, CH	8.09, m	129.8, CH	8.04, m
1″	176.1, C					
2″	34.2, CH	2.75, hep (7.0)				
3″, 4″	19.1, CH_3_	1.27, d (7.0)				

**Table 4:** NMR spectroscopic data (^1^H 400 MHz, ^13^C 100 MHz, CDCl_3_) of **7** and **7a**.

Position	7			7a	
	δ_C_, Type	δ_H_ (*J* in Hz)	HMBC	δ_C_, Type	δ_H_ (*J* in Hz)
1	126.2, C			132.3, C	
2	120.2, CH	6.66, brs	3, 4, 6, 7	126.7, CH	6.96, d (0.7)
3	149.8, C			145.3, C	
4	120.3, C			127.7, C	
5	113.0, CH	6.60, s	1, 3, 6, 8	121.8, CH	7.16, s
6	147.2, C			147.1, C	
7	15.7, CH_3_	2.17, brs	1, 2	16.1, CH_3_	1.63, brs
8	78.3, C			80.8, C	
9a	67.5, CH_2_	4.58, d(11.9)	4, 8, 10, 1″	62.9, CH_2_	4.96, d (11.4)
9b		4.51, d(11.9)	4, 8, 10, 1″		4.81, d (11.4)
10a	68.1, CH_2_	4.68 d(12.0)	1′, 4, 8, 9	63.6, CH_2_	5.12, d (11.4)
10b		4.65, d(12.0)	1’, 4, 8, 9		4.91, d (11.4)
1′	167.0, C			165.8, C	
2′	129.1, C			129.6, C	
3′	129.9, CH	7.98, m	1′, 5′, 7′	129.8, CH	7.95, m
4′	128.7, CH	7.41, m	3′, 5′, 7′	128.6, CH	7.43, m
5′	133.6, CH	7.55, tt (7.4,1.3)	3′, 7′	133.4, CH	7.55, tt (7.5, 1.3)
6′	128.7, CH	7.41, m	3′, 5′, 7′	128.6, CH	7.43, m
7′	129.9, CH	7.98, m	1′, 5′, 3′	129.8, CH	7.95, m
1″	177.9, C			176.3, C	
2″	34.1, CH	2.53, hep (7.0)	1″, 3″, 4″	34.0, CH	2.52, hep (7.0)
3″, 4″	18.9. 19.0, CH_3_	1.05, 1.08, d (7.0)	1″, 2″	18.8, 18.9, CH_3_	1.08, 1.10, d (7.0)
3-OCOCH_3_				21.3, 169.4, CH_3_, CO	2.38, s
6-OCOCH_3_				20.9, 168.8, CH_3_, CO	2.31, s
8-OCOCH_3_				21.4, 168.8, CH_3_, CO	2.00, s

**Table 5:** In vitro antiprotozoal activity and inhibition of hyperperistalsis of thymol derivatives.

Compound	IC_50_ μM (CI) ^a^		ID_50_ μmol/kg ± SD ^b^
	*Entamoeba histolytica*	*Giardia lamblia*	Inhibition of Hyperperistalsis
**1**	1.6 (1.8–1.57)	36.9 (38.3–5.9) *	2.000 ± 0.003 **
**1a**	0.84 (0.87–0.80)	24.2 (24.7–24.0) *	0.810 ± 0.021 **
**2**	169.6 (171.3–168.9) *	191.2 (192.1–190.8) *	0.457 ± 0.004 **
**3**	25.9 (26.2–25.7) *	48.3 (48.4–45.4) *	0.740 ± 0.003 **
**4**	61.2 (62.3–59.8) *	68.0 69.7–67.4) *	1.430 ± 0.006 **
**7**	45.6 (46.9–44.3) *	60.7 (62.3–56.9) *	0.380 ± 0.003 **
Pectolinaringenin	43.6 (44.9–41.9) *	68.7 (70.3–67.4) *	0.598 ± 0.001 **
**9** ^c^	184.9 (186.9–180.7) *	167.4 (168.7–165.8) *	0.85 ± 0.005
Emetine ^d^	2.18 (2.2–2.14)	0.83 (0.87–0.82)	
Metronidazole ^d^	0.23 (0.58–0.17)	1.22 (1.57–0.81)	
Quercetin	-	-	1.1 ± 0.001
Loperamide hydrochloride ^d^	-	-	0.2 ± 0.001

^a^ Results are expressed as mean (*n* = 6), CI = 95% confidence intervals; * *p* < 0.05 compared to emetine and metronidazole; ^b^ Results are expressed as mean (*n* = 6) ± SD; ** *p* < 0.05 compared to loperamide hydrochloride. ^c^ See ref. 7; ^d^ Positive controls.

## Supplementary Materials

Supplementary materials can be accessed at: http://www.mdpi.com/1420-3049/21/9/1132/s1.
