# Supplementary material for: Antidiarrheal Thymol Derivatives from Ageratina glabrata. Structure and Absolute Configuration of 10-Benzoyloxy-8,9-epoxy-6-hydroxythymol Isobutyrate
Source: Molecules. 2016 Sep 12;21(9):1132. doi: 10.3390/molecules21091132 (PMC6274089; doi:10.3390/molecules21091132)
Supplement: Supplementary file 1 [file molecules-21-01132-s001.pdf]

Antidiarrheal Thymol Derivatives from *Ageratina glabrata*. Structure and Absolute Configuration of 10-Benzoyloxy-8,9-epoxy-6-hydroxythymol Isobutyrate

Celia Bustos-Brito, Valeria J. Vázquez-Heredia, Fernando Calzada, Lilian Yépez-Mulia, José S. Calderón, Simón Hernández-Ortega, Baldomero Esquivel, Normand García-Hernández and Leovigildo Quijano

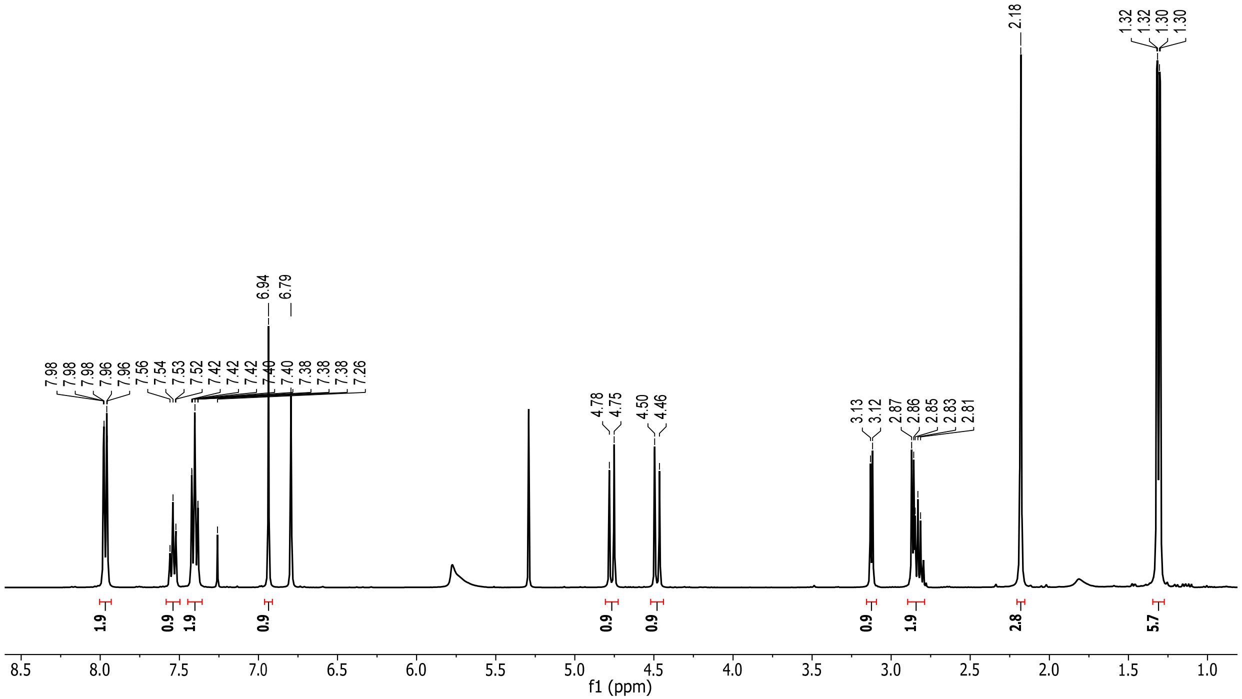

Figure S1. ^{1}H-NMR ($CDCl_3$, 400 MHz) spectrum of **1**.

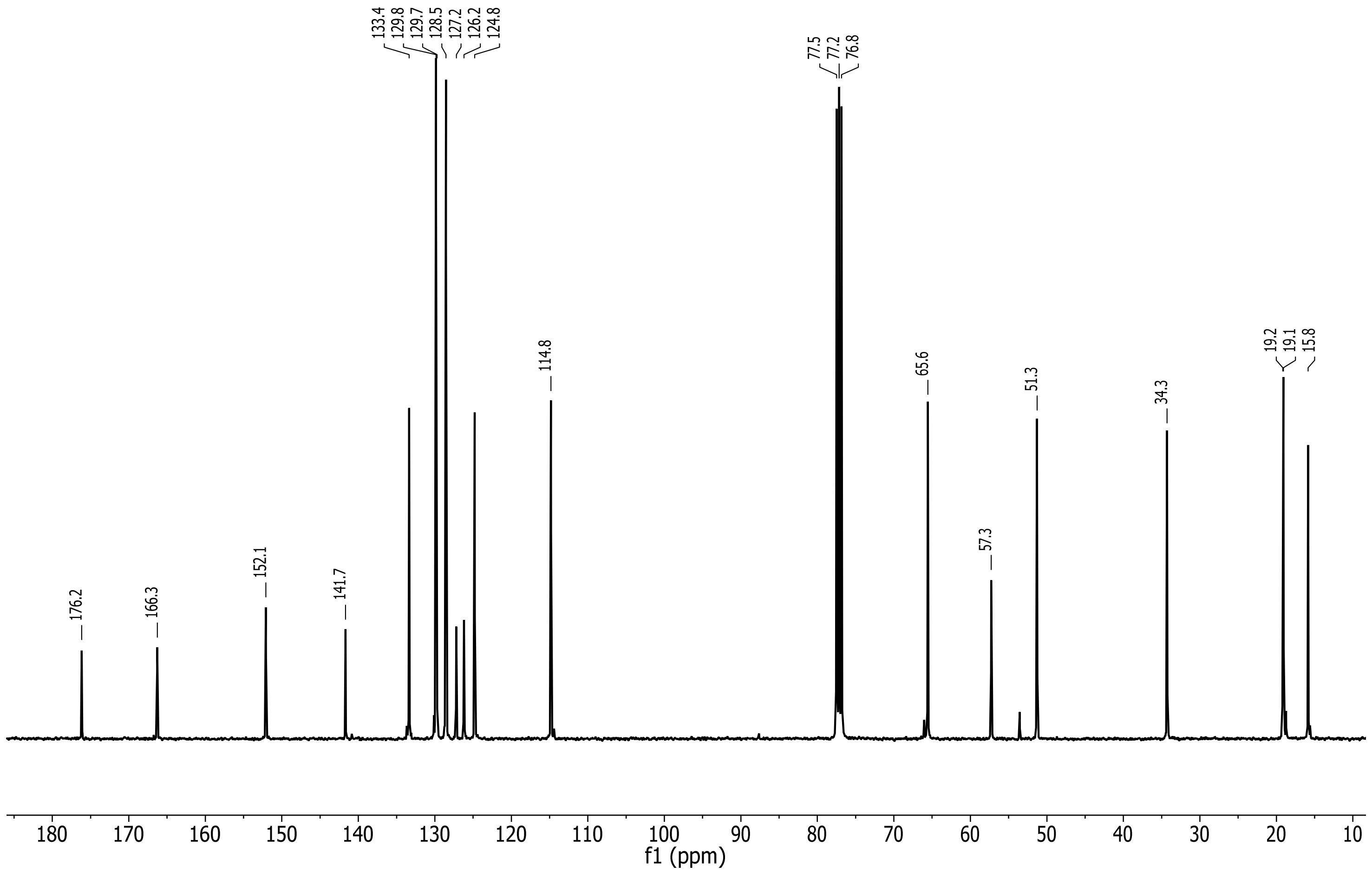

Figure S2. ^{13}C-NMR ($CDCl_3$, 100 MHz) spectrum of **1**.

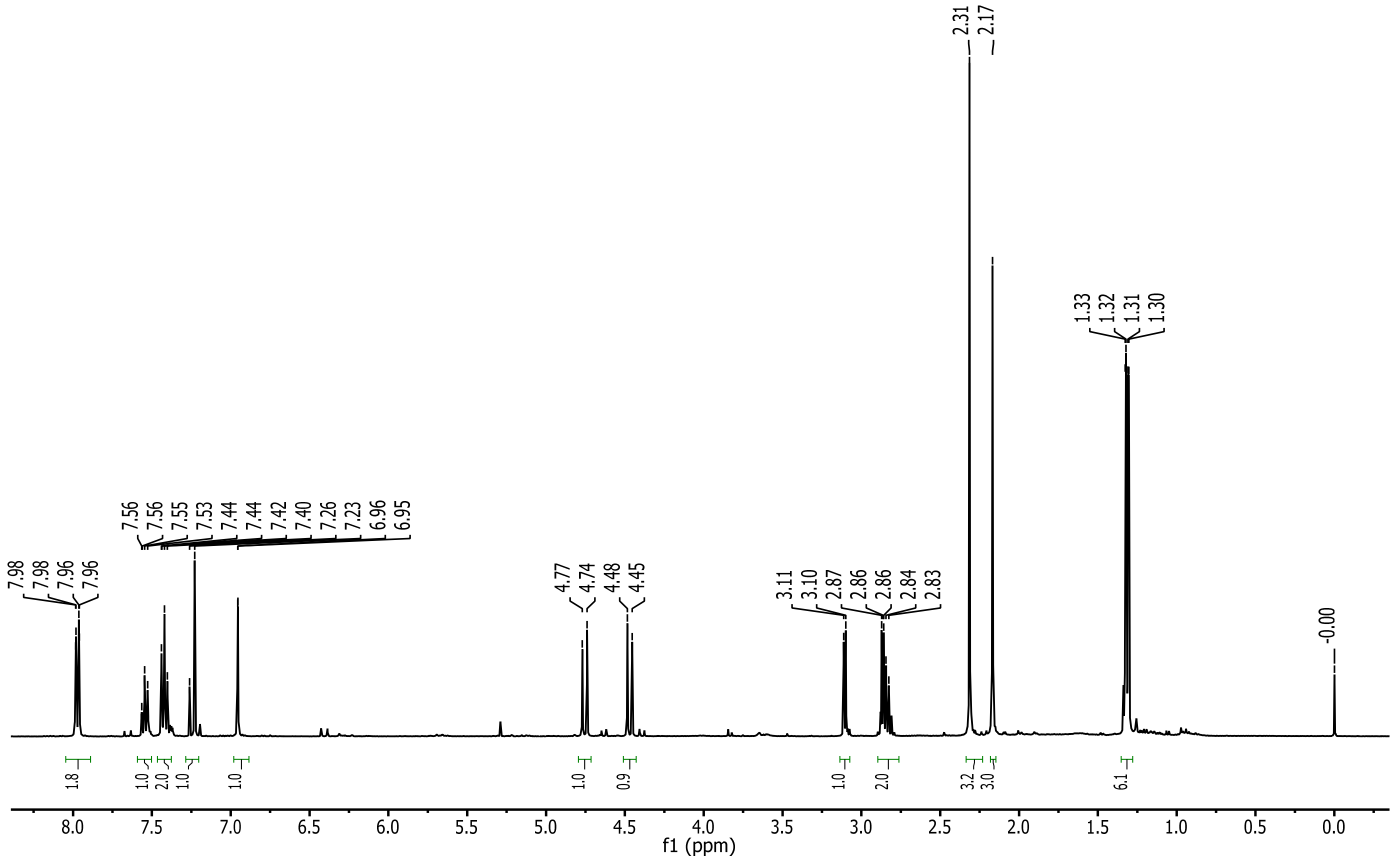

Figure S3. ^{1}H-NMR ($CDCl_3$, 400 MHz) spectrum of **1a**.

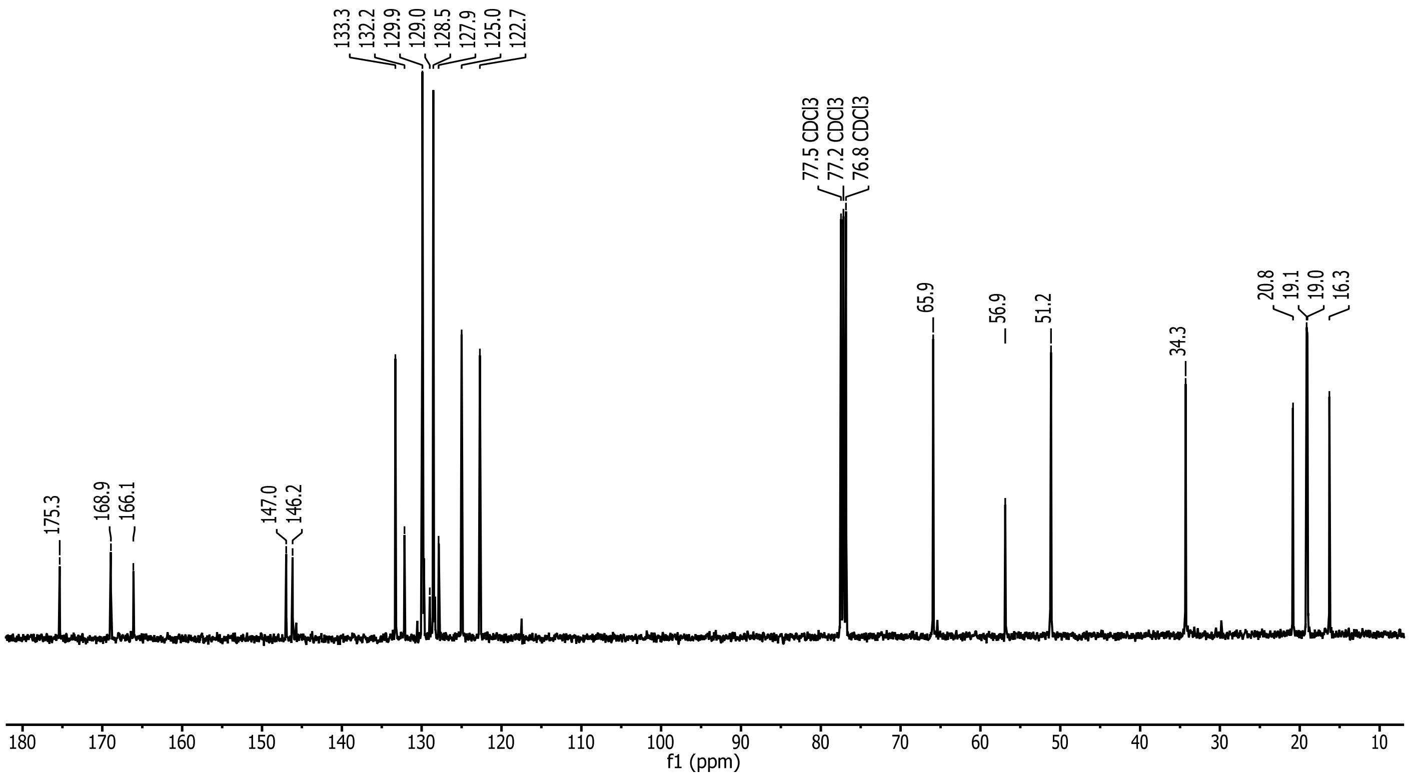

Figure S4. ^{13}C-NMR ($CDCl_3$, 100 MHz) spectrum of **1a**.

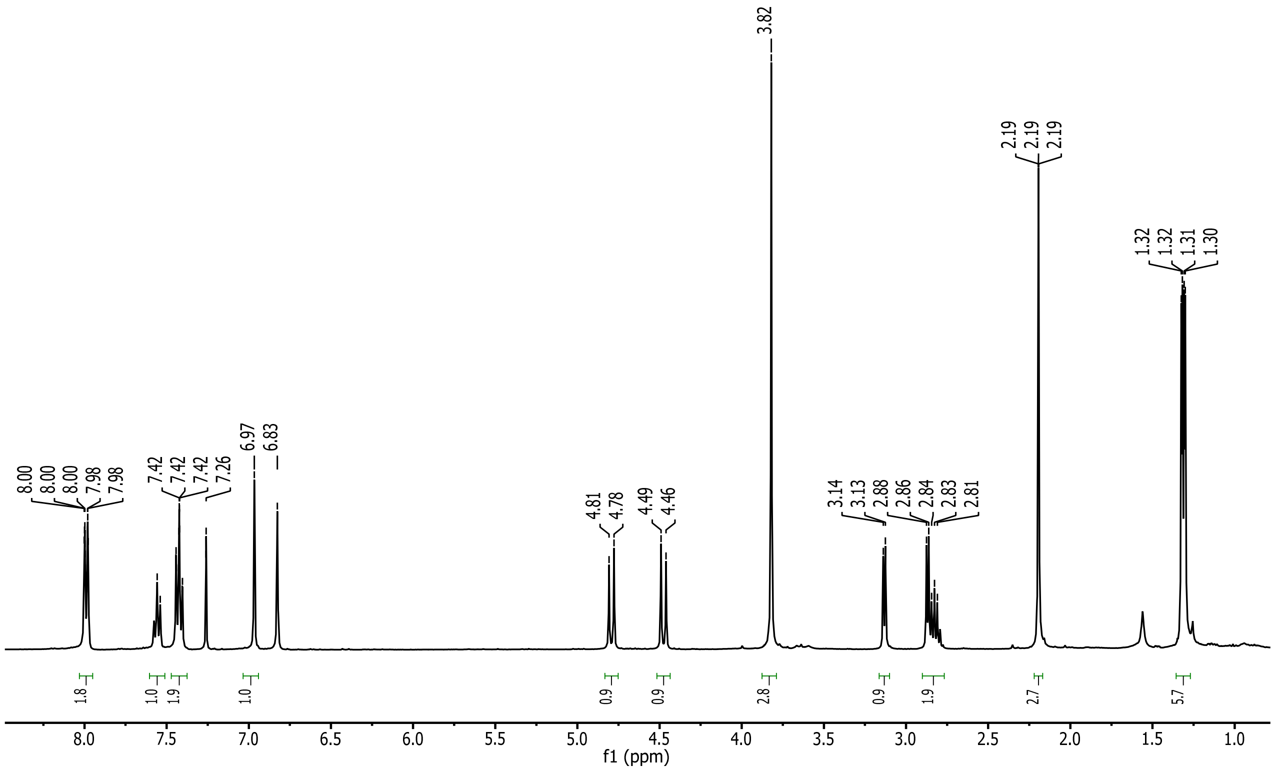

Figure S5. ^{1}H-NMR ($CDCl_3$, 400 MHz) spectrum of **2**.

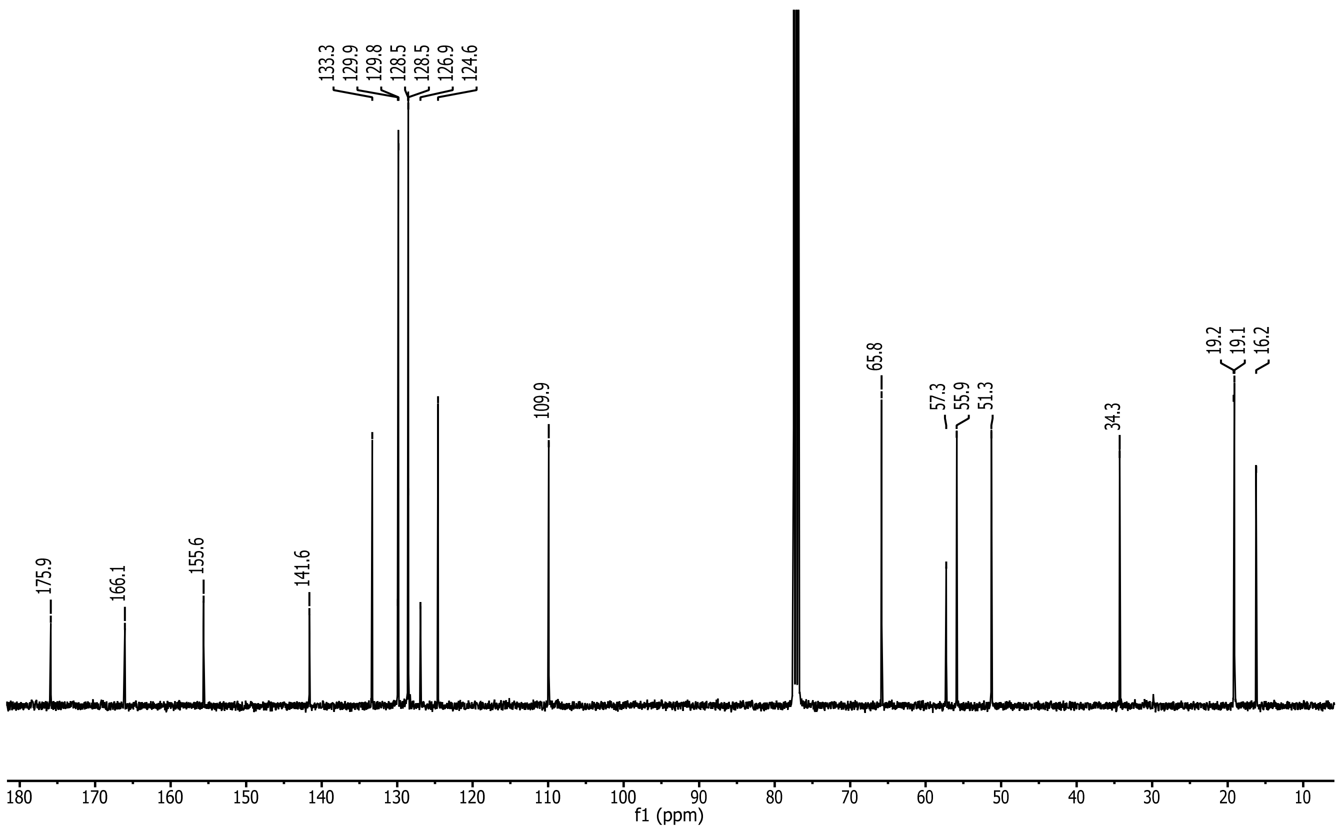

Figure S6. ^{13}C-NMR ($CDCl_3$, 100 MHz) spectrum of **2**.

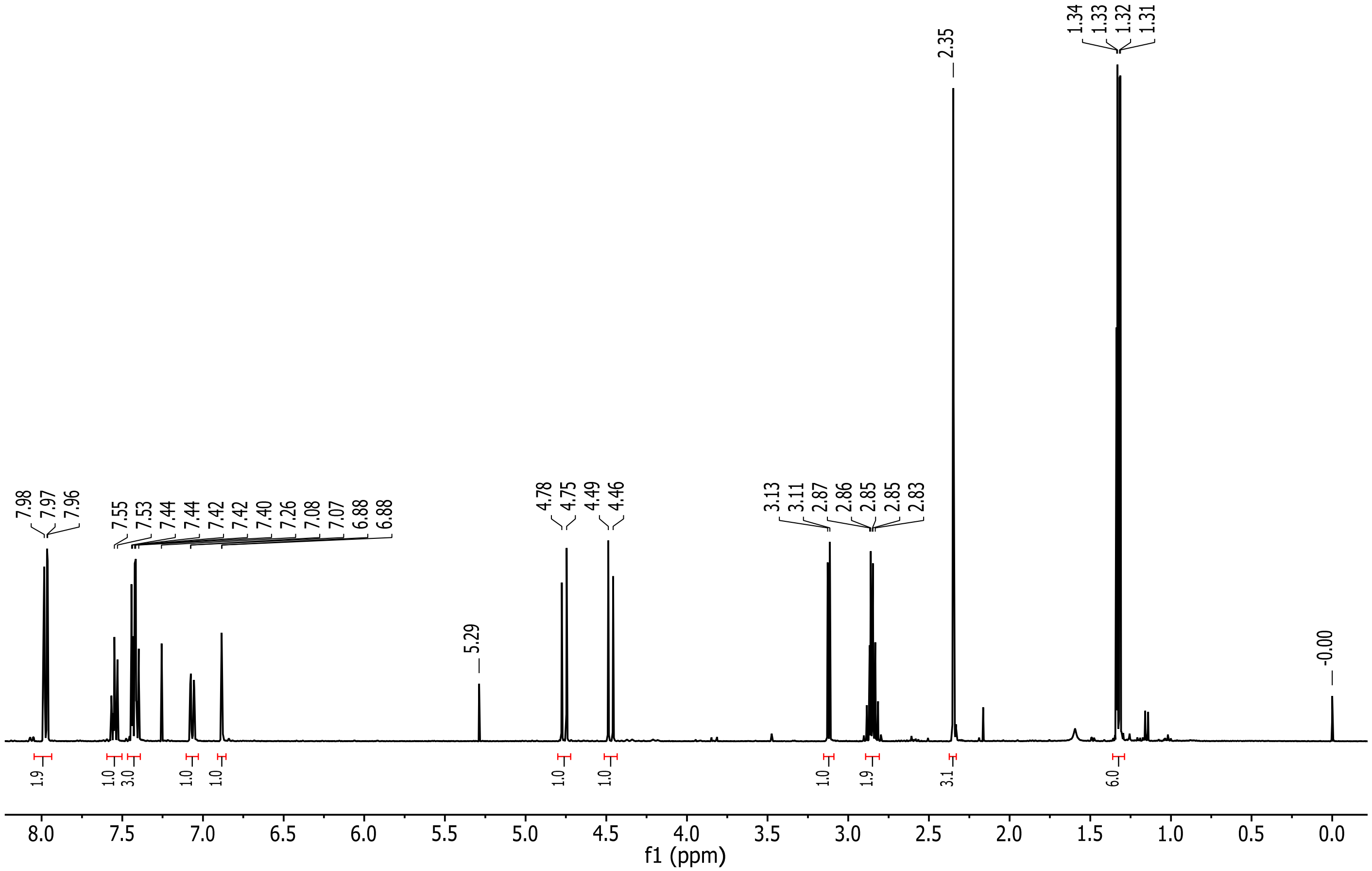

Figure S7. ^{1}H-NMR ($CDCl_3$, 400 MHz) spectrum of **3**.

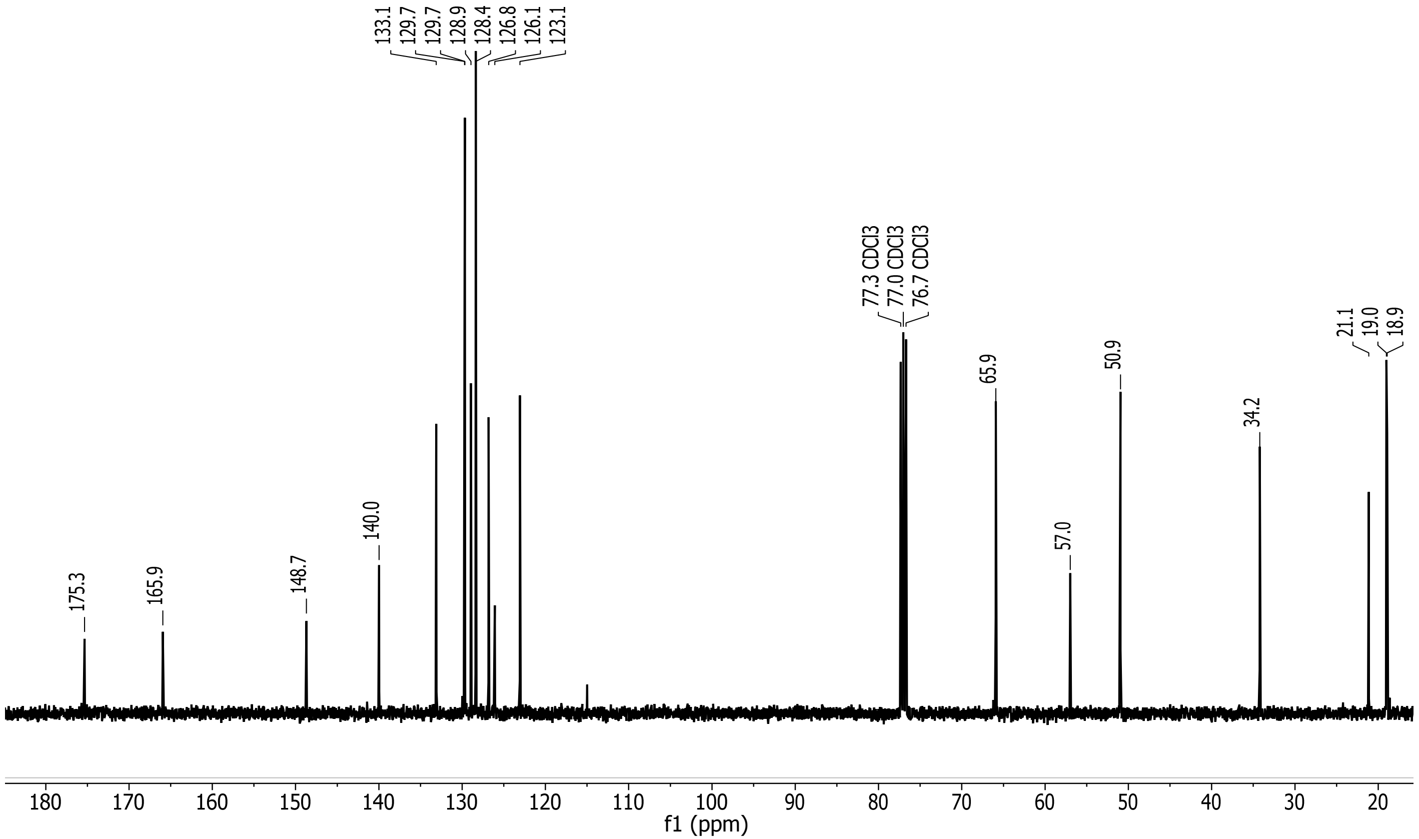

Figure S8. ^{13}C-NMR ($CDCl_3$, 100 MHz) spectrum of **3**.

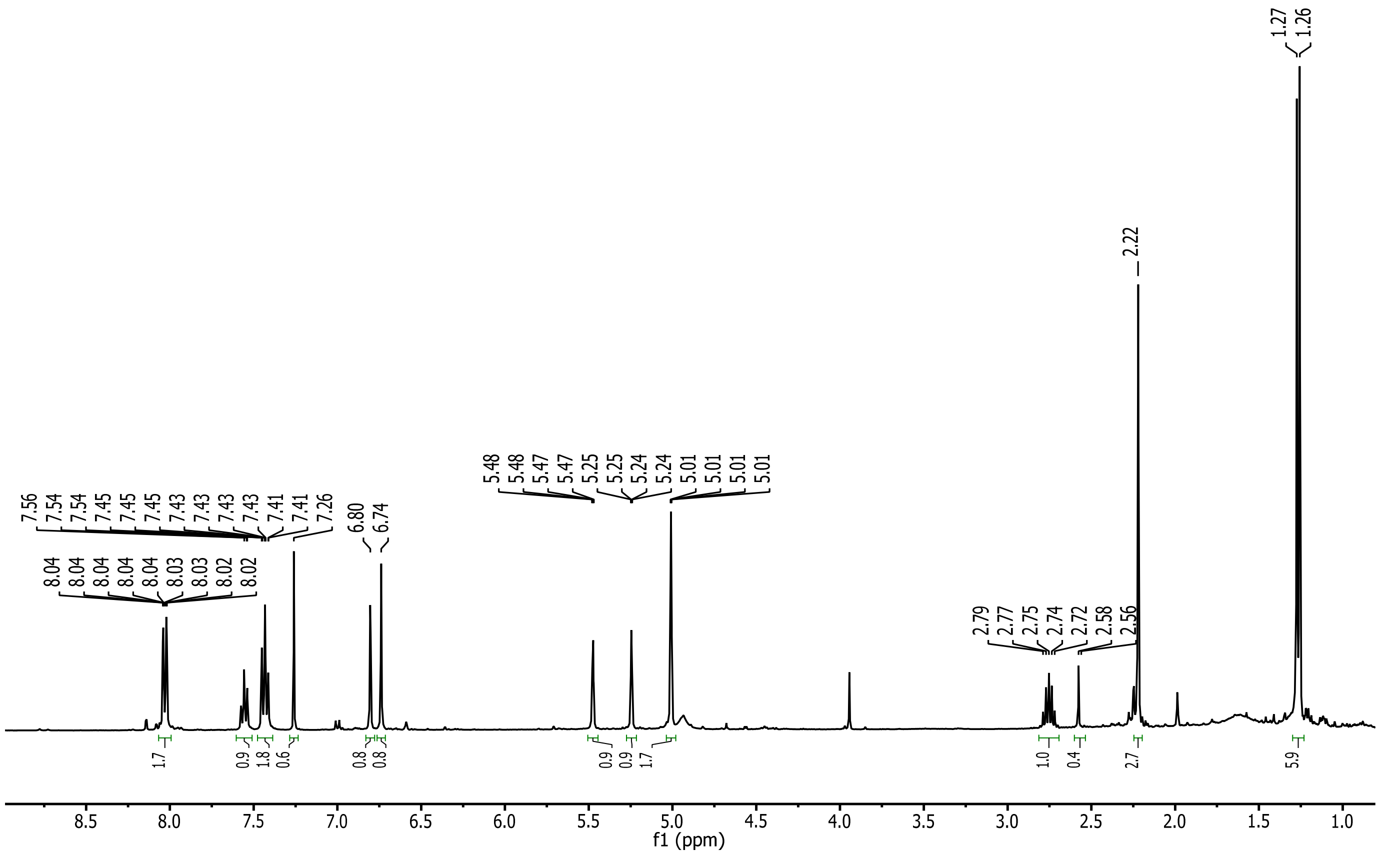

Figure S9. ^{1}H-NMR ($CDCl_3$, 400 MHz) spectrum of **4**.

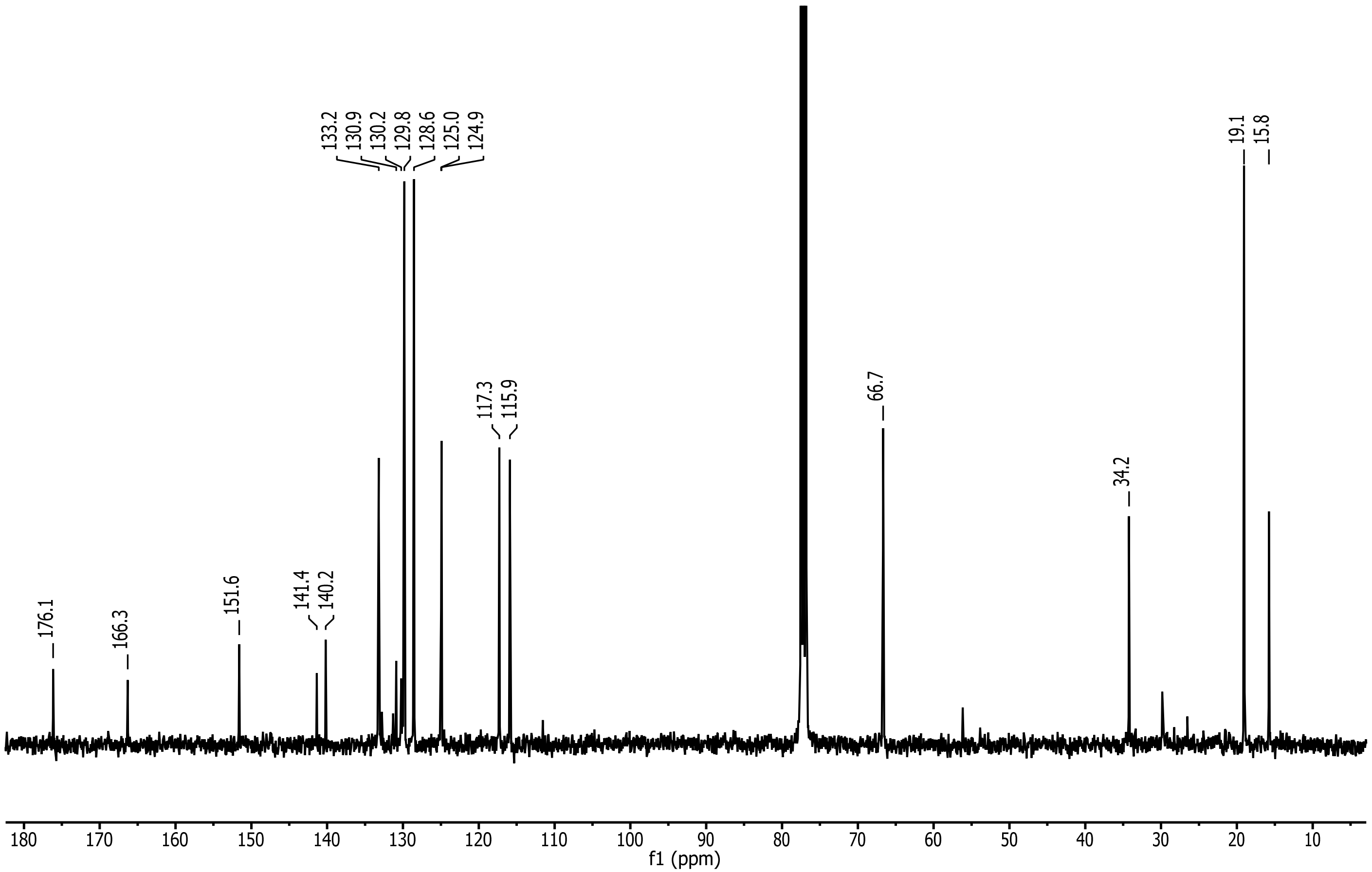

Figure S10. ^{13}C-NMR ($CDCl_3$, 100 MHz) spectrum of **4**.

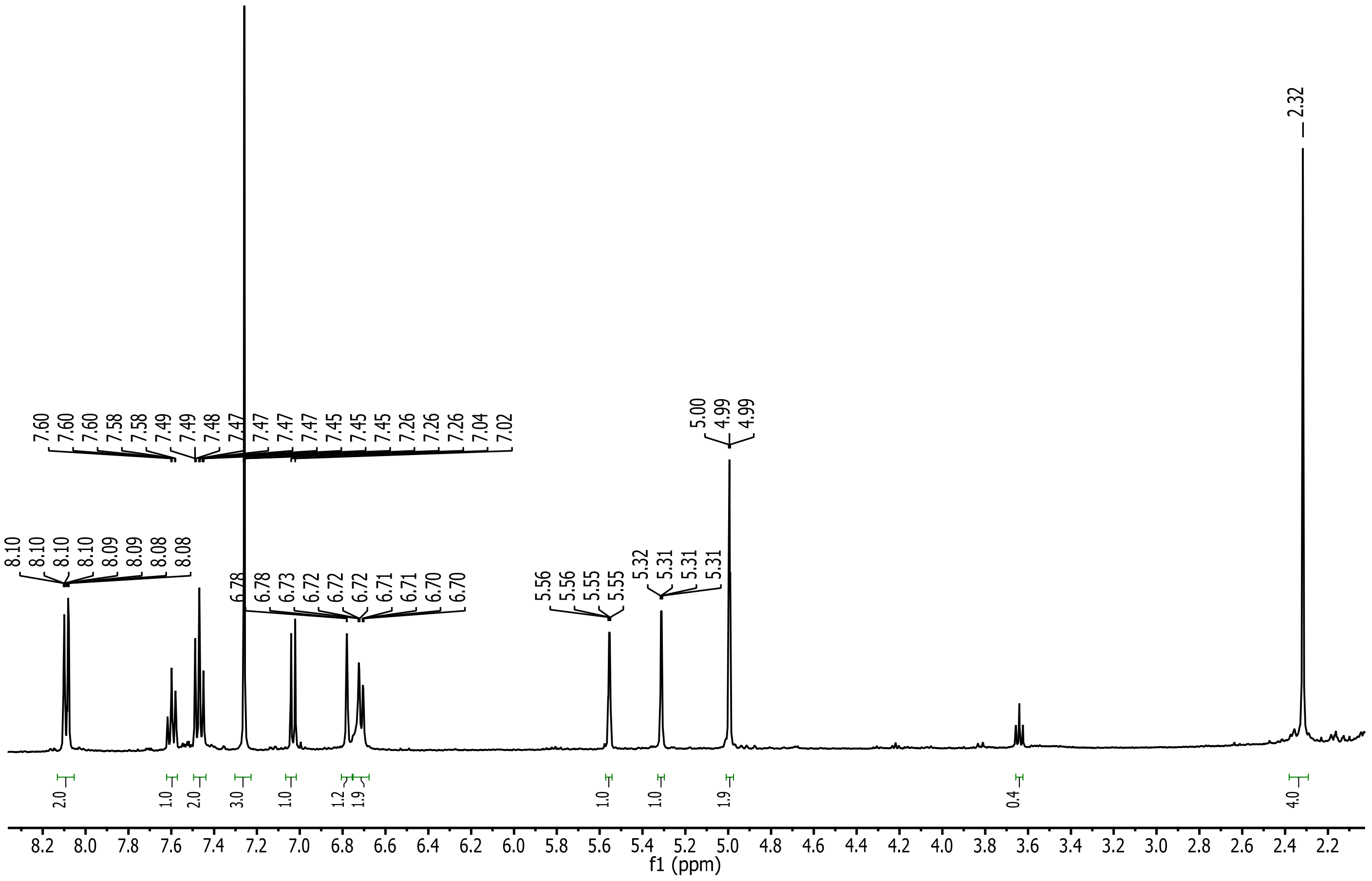

Figure S11. ^{1}H-NMR ($CDCl_3$, 400 MHz) spectrum of **5**.

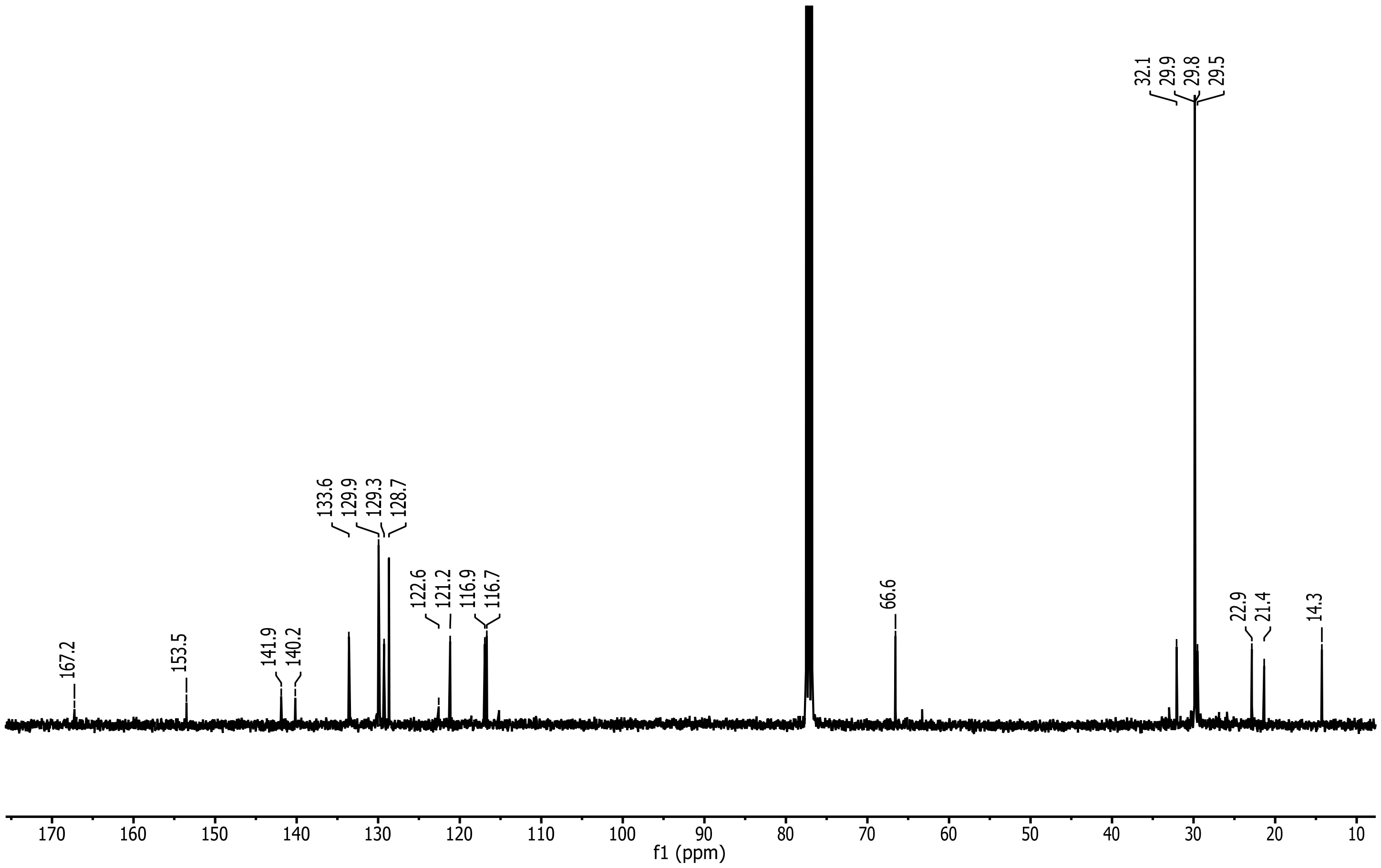

Figure S12. ^{13}C-NMR ($CDCl_3$, 100 MHz) spectrum of **5**.

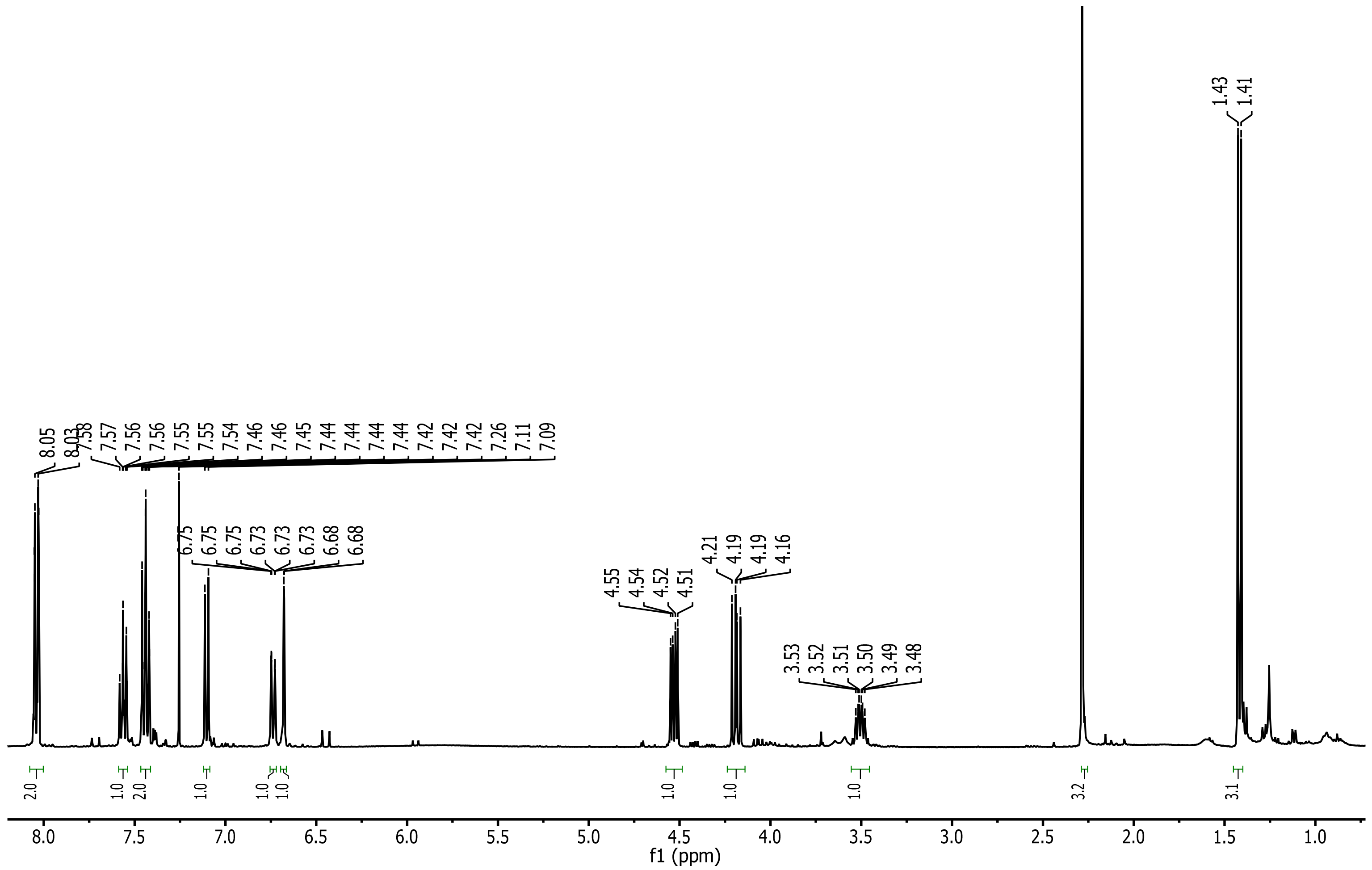

Figure S13. ^{1}H-NMR ($CDCl_3$, 400 MHz) spectrum of **6**.

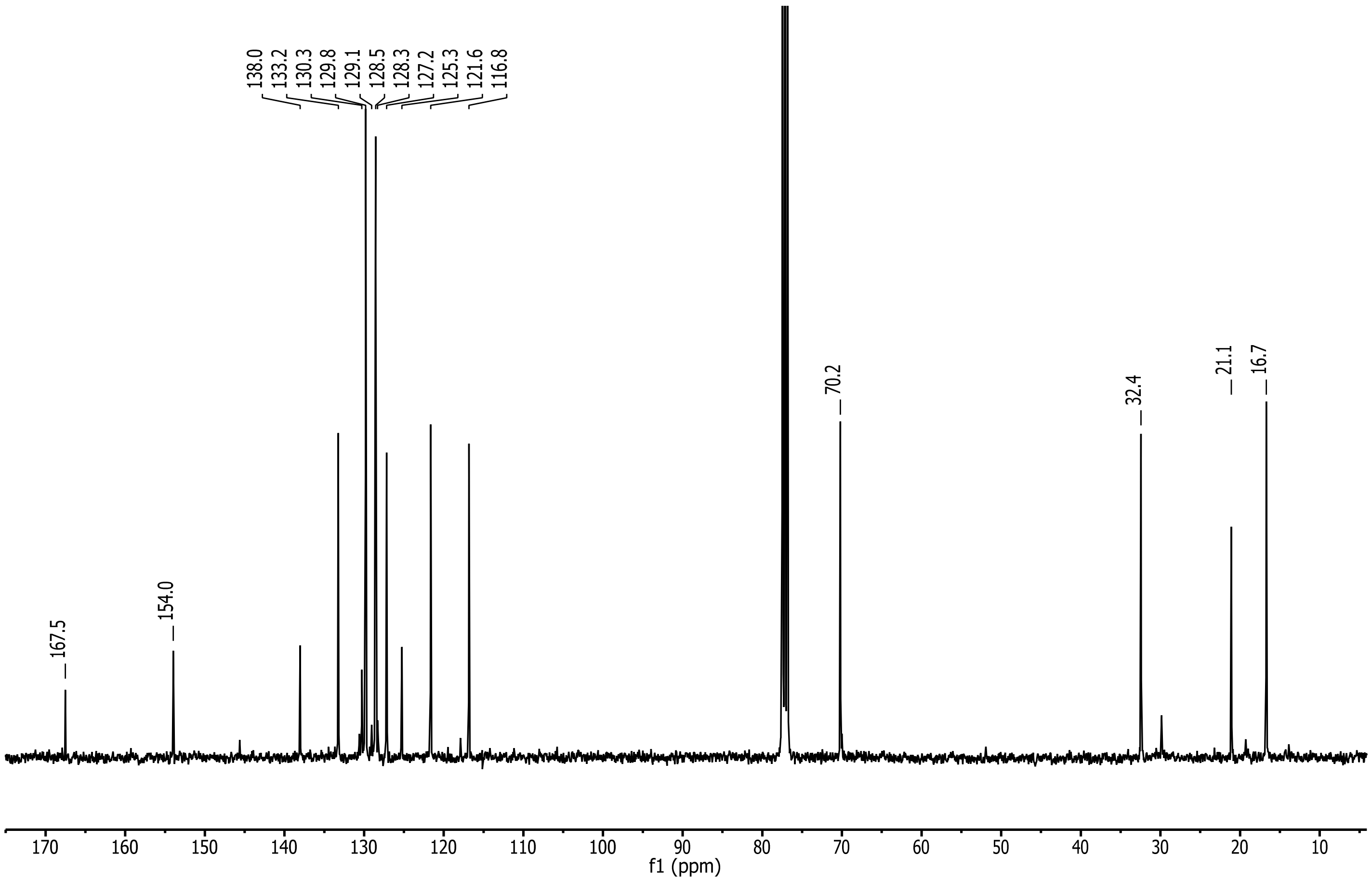

Figure S14. ^{13}C-NMR ($CDCl_3$, 100 MHz) spectrum of **6**.

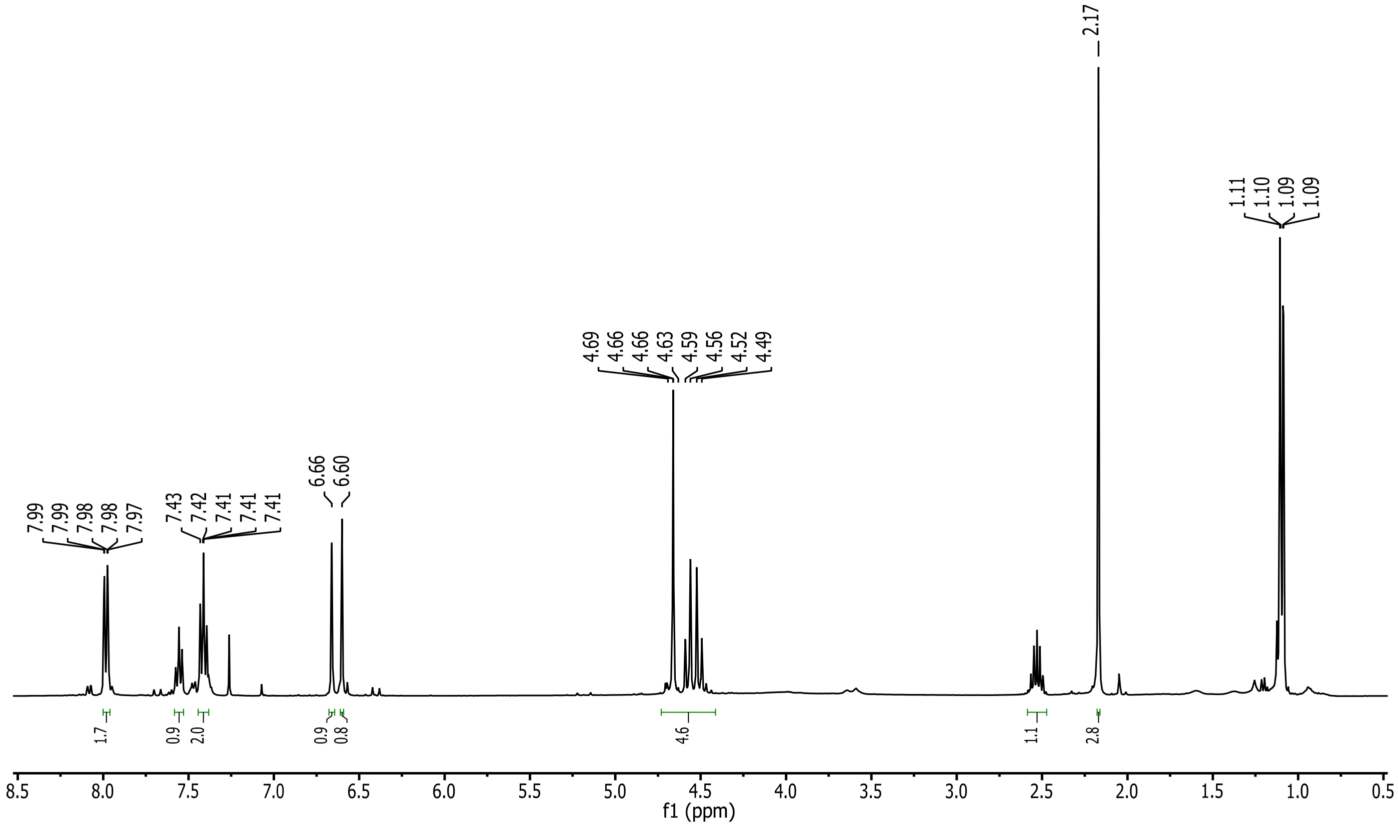

Figure S15. ^{1}H-NMR ($CDCl_3$, 400 MHz) spectrum of **7**.

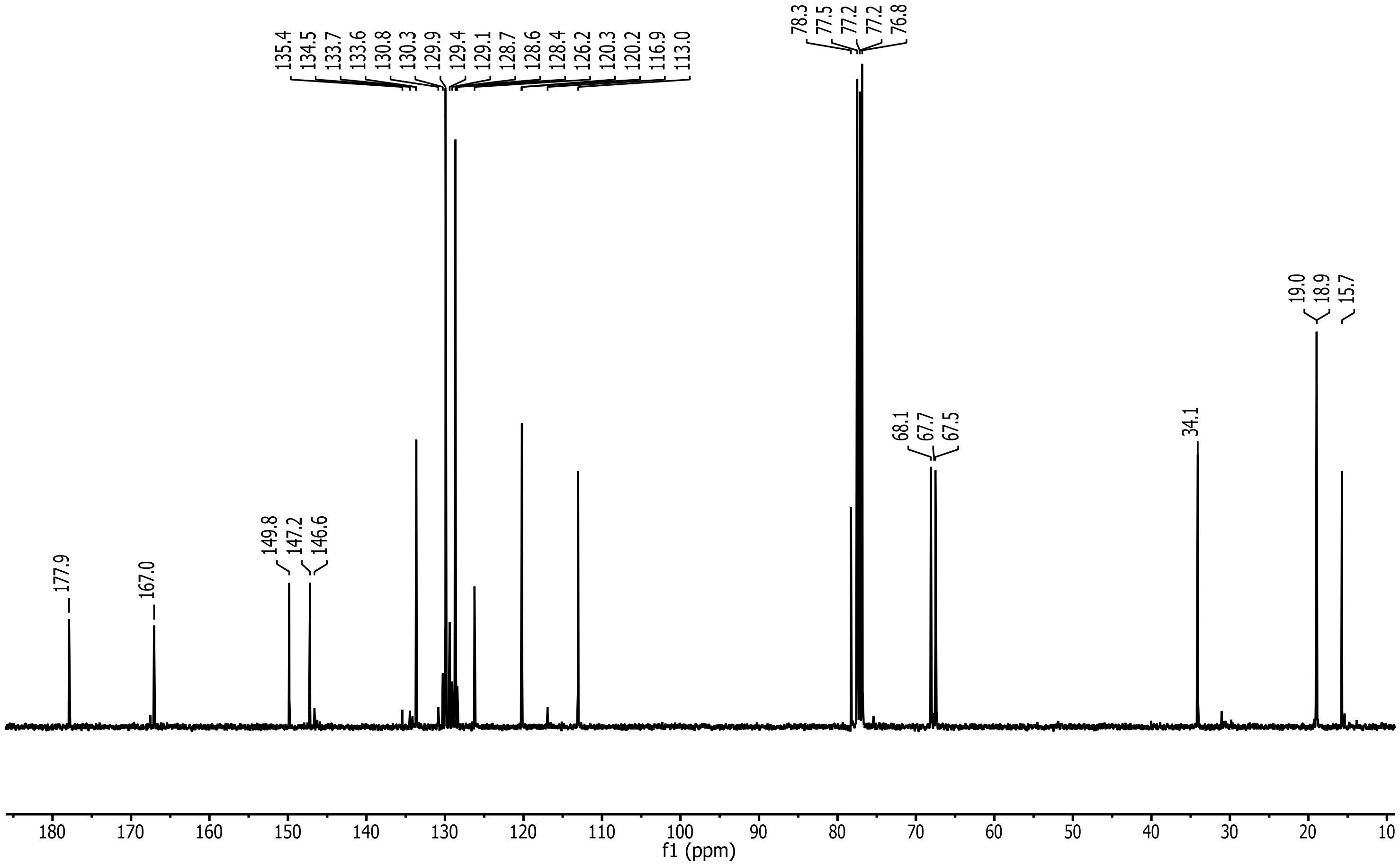

Figure S16. ^{13}C-NMR ($CDCl_3$, 100 MHz) spectrum of **7**.

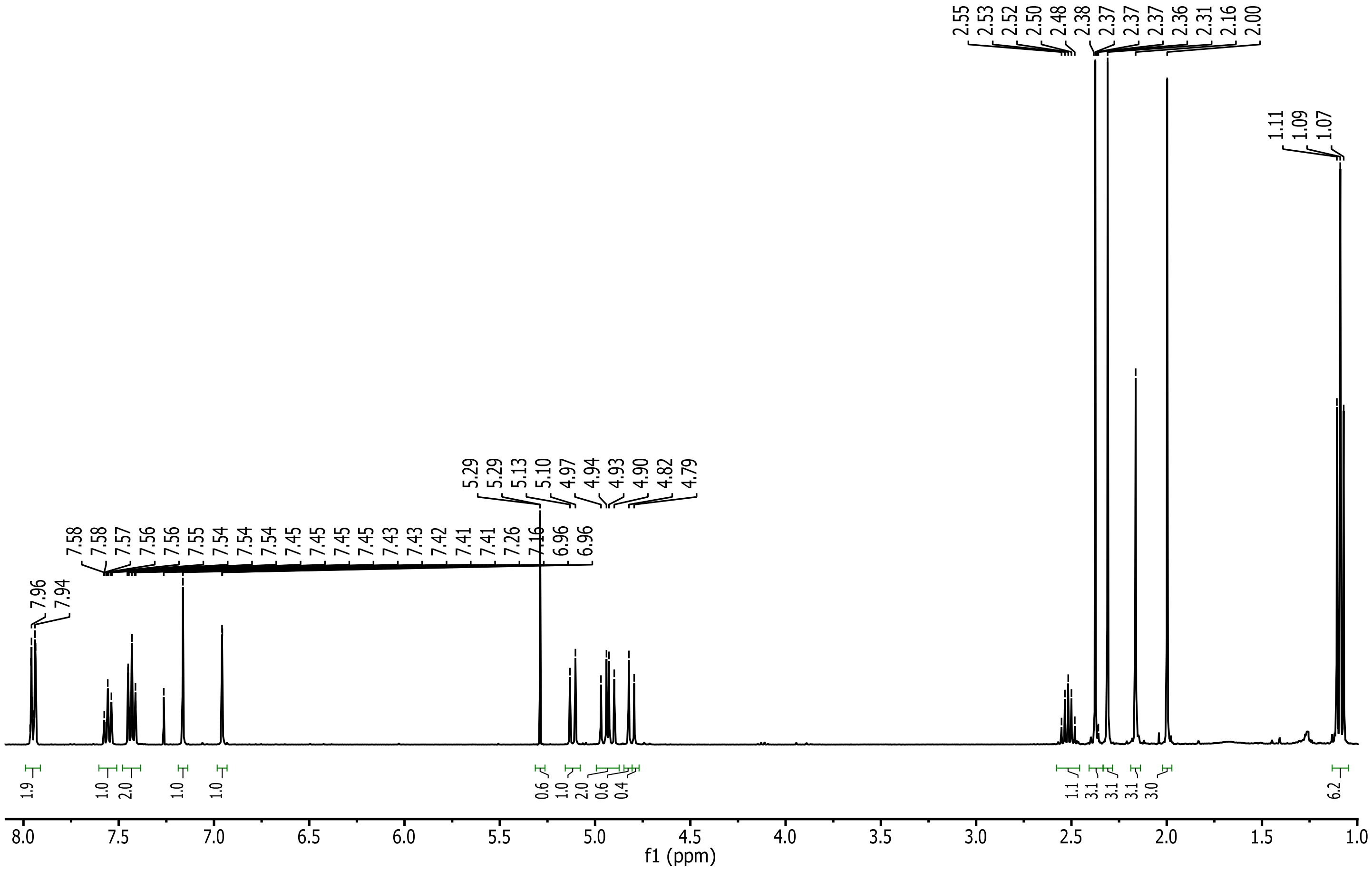

Figure S17. ^{1}H-NMR ($CDCl_3$, 400 MHz) spectrum of **7a**.

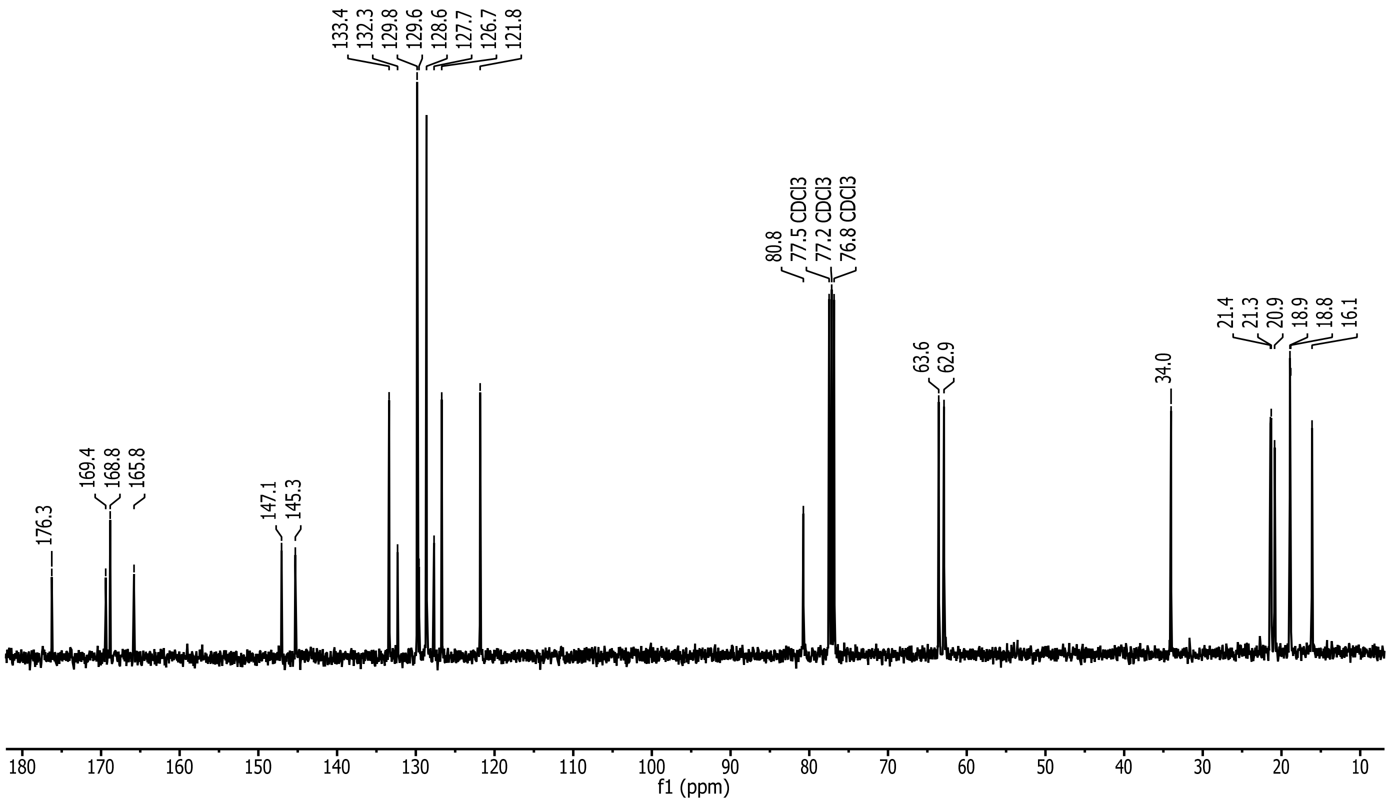

Figure S18. ^{13}C-NMR ($CDCl_3$, 100 MHz) spectrum of **7a**.

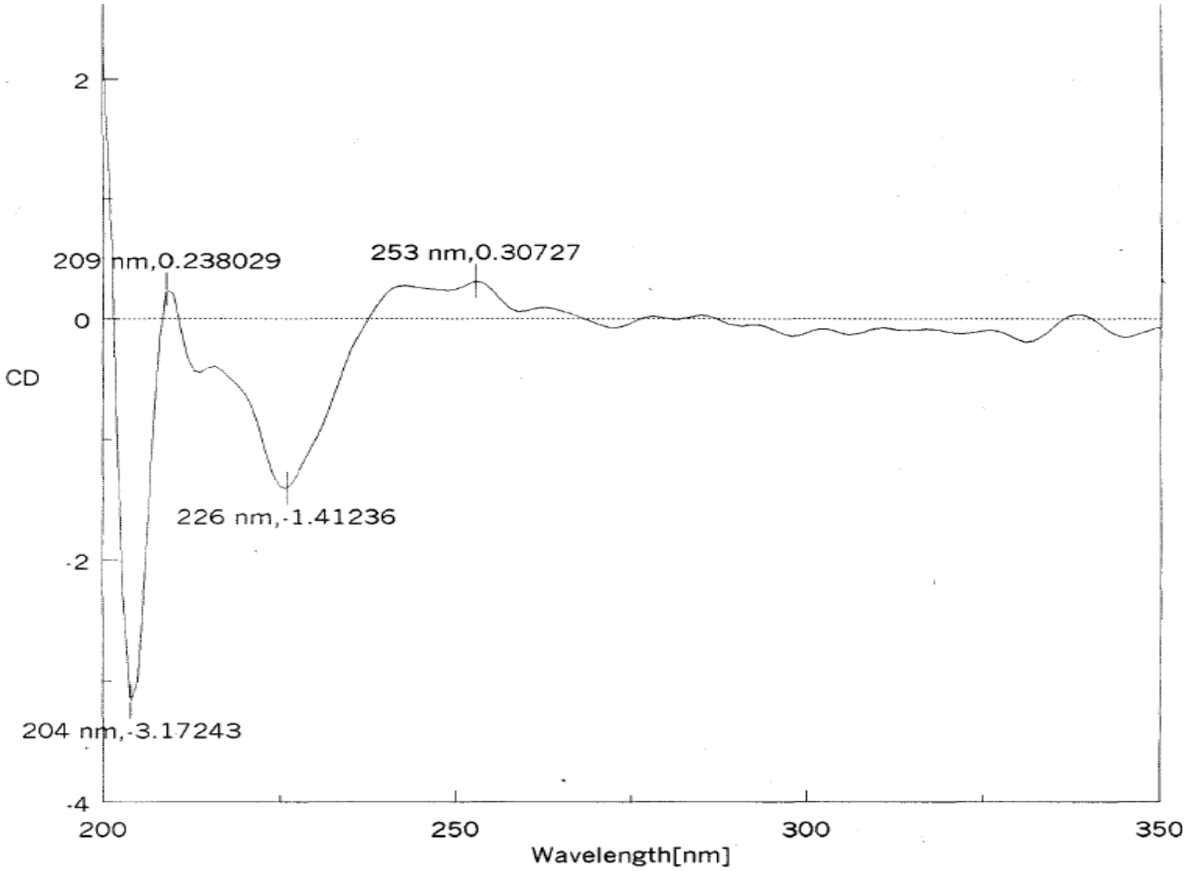

Figure S19. ECD spectrum of **1**.

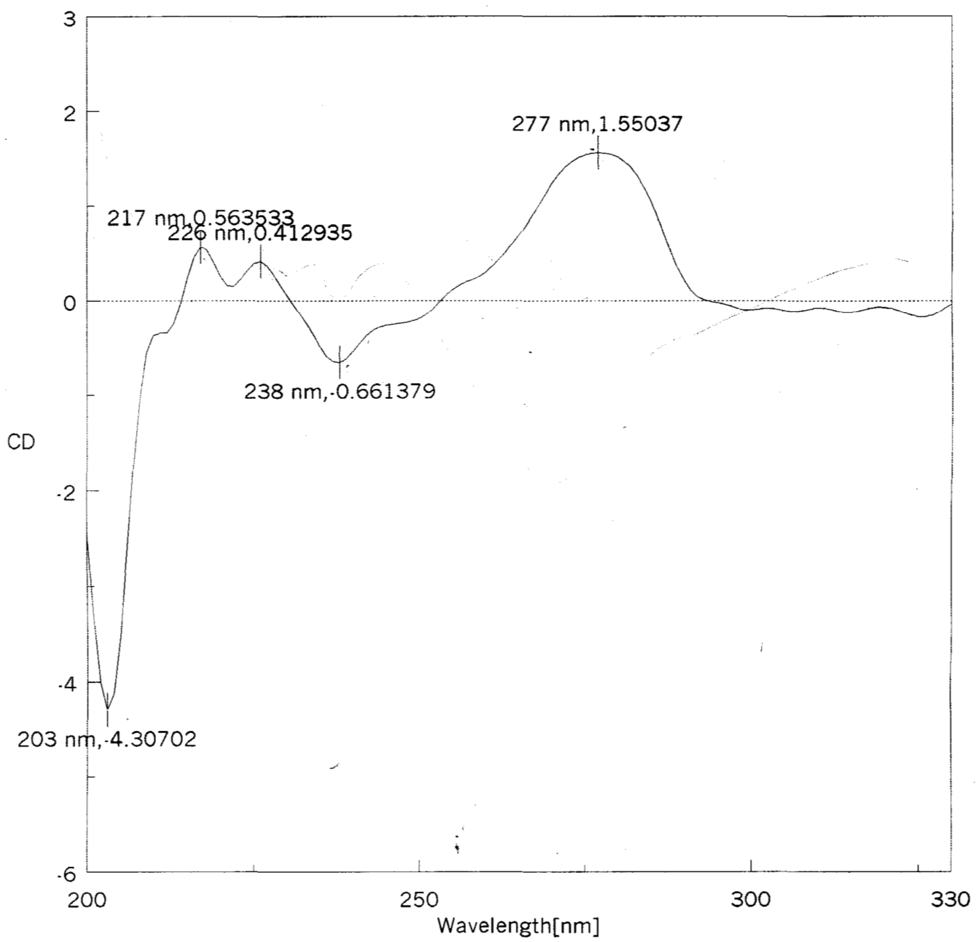

Figure S20. ECD spectrum of **6**.

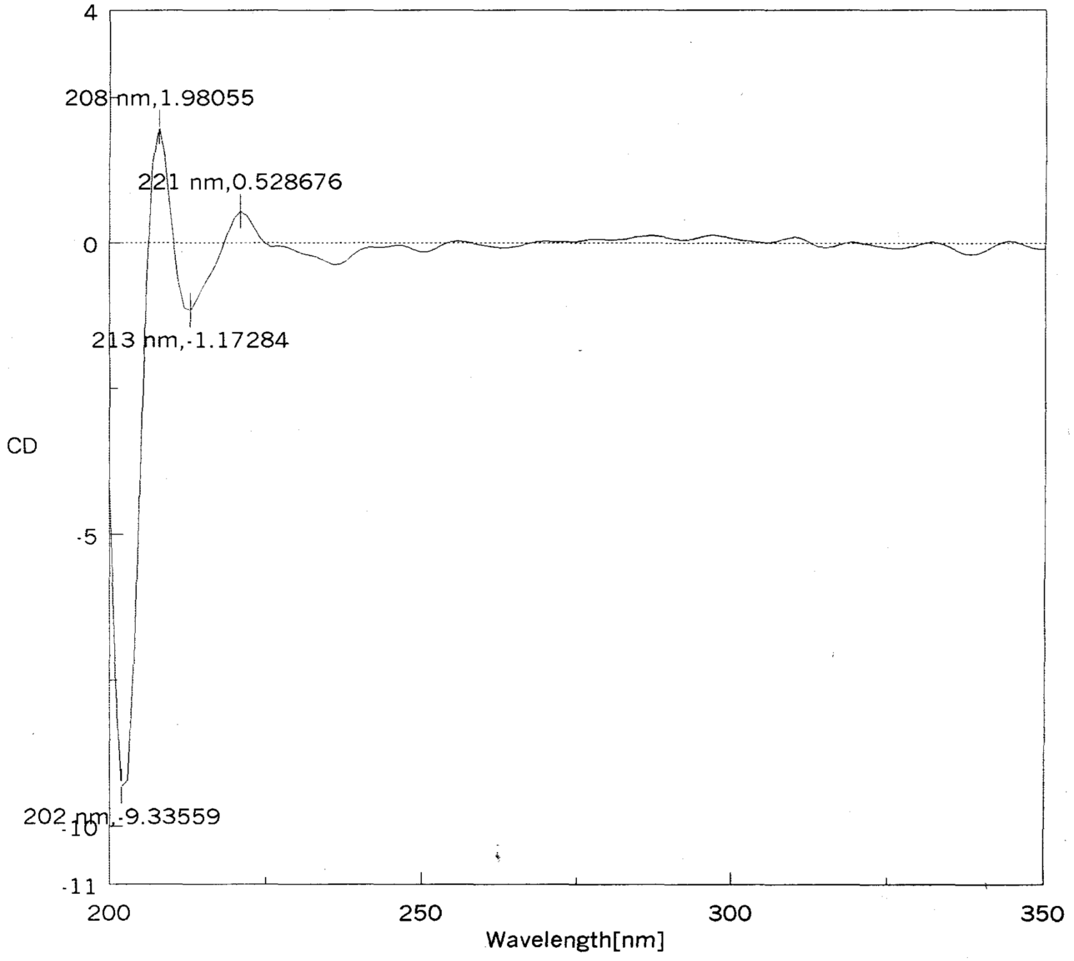

Figure S21. ECD spectrum of **7**.
